# Enhanced CO_2_ adsorption *via* one-step thermochemical activation of Alfa fiber

**DOI:** 10.1039/d6ra06467a

**Published:** 2026-08-26

**Authors:** Hicham Akaya, Zineb Ouzrour, Badr El Aalami, Alexandre Babin, Hosni Idrissi, Xiaotao Bi, Tausif Altamash, Johan Jacquemin

**Affiliations:** a College of Chemical Sciences and Engineering (CCSE), Department of Materials Science, Energy and Nano-engineering (MSN), Mohammed VI Polytechnic University (UM6P) 43150 Benguerir Morocco tausif.altamash@um6p.ma johan.jacquemin@um6p.ma; b High Throughput Multidisciplinary Research Laboratory (HTMR), College of Chemical Sciences and Engineering (CCSE), Mohammed VI Polytechnic University (UM6P) Lot 660 Benguerir 43150 Morocco; c Institute of Mechanics, Materials and Civil Engineering, IMAP Division, UCLouvain Place Sainte Barbe, 2 B-1348 Louvain-la-Neuve Belgium; d Chemical and Biological Engineering Department, University of British Columbia Vancouver British Columbia V6T 1Z3 Canada

## Abstract

In this work, Moroccan Alfa fibers were converted into porous biochars through a one-step thermochemical activation using KOH, ZnCl_2_, and melamine at impregnation ratios of 0.5, 1.0, and 1.5. The influence of the activating agents on the physicochemical properties and CO_2_ adsorption behavior of the resulting materials was systematically investigated using structural and surface characterization techniques. Chemical activation substantially enhanced pore development compared with the non-activated biochar, with KOH producing the most developed hierarchical micro–mesoporous structure. Among all samples, Af-K1 exhibited the highest CO_2_ adsorption capacity, reaching 3.70 mmol g^−1^ at 30 °C and 1 bar, which increased to 5.26 mmol g^−1^ at 0 °C. This superior performance was primarily attributed to its larger micropore volume and optimized pore-size distribution. Af-K1 also demonstrated excellent regeneration stability, retaining approximately 99% of its initial adsorption capacity after ten adsorption–desorption cycles. Furthermore, the material exhibited an IAST-predicted CO_2_/N_2_ selectivity of 58 for a simulated flue gas mixture (15% CO_2_), while model extrapolation suggested a selectivity of 24–41 under direct air capture (DAC) conditions (400 ppm CO_2_). These predicted DAC values should be regarded as indicative and require experimental validation under low-pressure and mixed-gas conditions. Thermodynamic analysis indicated that CO_2_ adsorption is spontaneous and exothermic, with Δ*H*° = −13.19 kJ mol^−1^ and Δ*S*° = −0.041 kJ mol^−1^ K^−1^, while Δ*G*° remained negative (−2.11 to −0.28 kJ mol^−1^) over the investigated temperature range, confirming the thermodynamic favorability of the adsorption process. These findings demonstrate that one-step KOH activation provides an efficient and sustainable strategy for producing high-performance biochar adsorbents for CO_2_ capture and separation.

## Abstract 2

One-step thermochemical activation of Moroccan Alfa fiber with KOH, ZnCl_2_, and melamine reveals the key role of activation chemistry in tailoring porosity and CO_2_ adsorption, with KOH-activated biochar exhibiting the highest adsorption performance.

## Graphical abstract

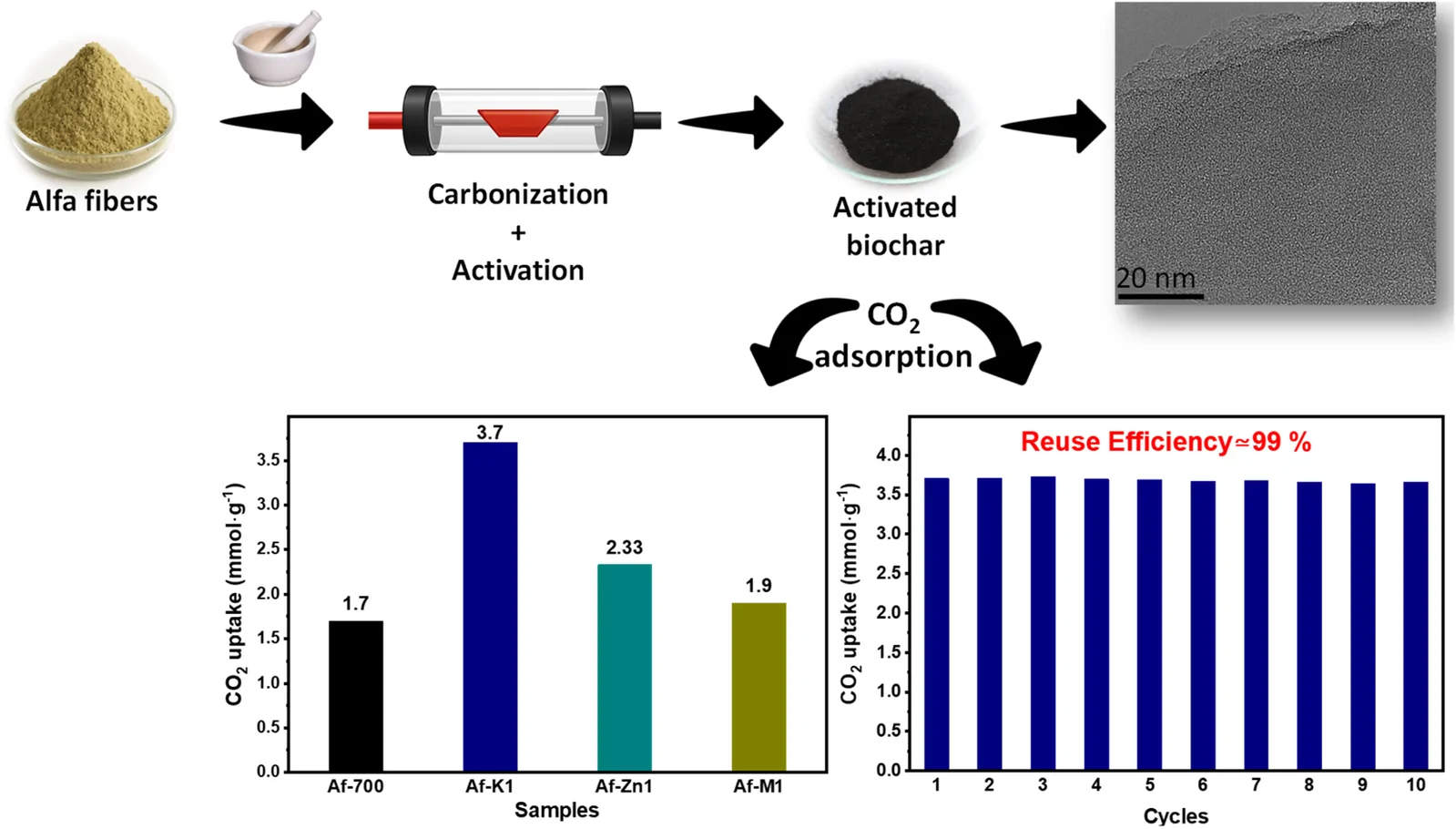

## 1. Introduction

The pressing need to mitigate rising atmospheric carbon dioxide (CO_2_) levels has intensified research and development of efficient carbon capture technologies.^1^ Global atmospheric CO_2_ concentrations have risen from approximately 280 ppm during the pre-industrial era to more than 420 ppm at present, contributing to an increase of approximately 1.1 °C in the global mean temperature since the late nineteenth century.^2^ This warming trend, primarily driven by anthropogenic activities, has been associated with more frequent extreme weather events, sea-level rise, and widespread ecological disturbances. Achieving the 1.5 °C target established by the Paris Agreement requires not only substantial reductions in greenhouse gas emissions but also the active removal of CO_2_ already accumulated in the atmosphere.^3^

According to the International Energy Agency (IEA) Net Zero Emissions Scenario for 2050, direct air capture technologies are anticipated to remove over 85 million tonnes of CO_2_ annually by 2030, escalating to approximately 980 million tonnes by 2050.^4^ This represents a substantial expansion requiring rapid technological advancement from the current capture capacity of approximately 0.01 million tonnes per year. Among various approaches, direct air capture (DAC) has emerged as a key technology for delivering negative emissions, as it enables CO_2_ removal directly from the atmosphere regardless of the emission source.^5^ However, the success of DAC relies on the availability of high-performance, economically viable adsorbents capable of operating under ambient conditions, enabling large-scale CO_2_ removal to help counteract continued atmospheric greenhouse gas accumulation.^6^ There are two types of materials used: liquids, such as amine solutions,^7^ which have disadvantages due to their corrosive nature and the need for huge energy for the desorption process.^8^ The solid materials, such as metal–organic frameworks,^9^ and zeolites^10^ offer high porosity and can be customized for better performance.^11^ However, biochar is an alternative solid material obtained from natural resources, which has gained significant attention for its renewability, tunable surface properties, and potential for large-scale deployment.^12^

Historically, biochar has been widely used in agriculture to enhance soil fertility, increase water-holding capacity, and promote carbon storage.^13^ Nevertheless, the inherent porosity and surface chemistry of biochar also make it an attractive low-cost adsorbent for CO_2_ capture applications.^14^ Previous studies have shown that the adsorption performance of biochar can be improved through different physical and chemical activation and surface modification strategies that tailor the pore structure and introduce additional functional groups, thereby enhancing its adsorption properties. For example, Ma *et al.*^15^ enhanced the porosity and nitrogen-functionalized surfaces of sawdust-derived biochar by incorporating urea phosphate and demonstrated the mechanisms of carbonization, activation, and nitrogen doping to produce N-doped porous carbon. Sarwar *et al.*^16^ also investigated chemically activated corncob hydrochar using H_2_PO_2_, ZnCl_2_, and KOH to improve CO_2_ uptake under elevated pressures (up to 16 bar) over a wide temperature range. Similarly, Ding *et al.*^17^ prepared seaweed-derived porous biochars *via* one-step KOH activation and demonstrated that the developed microporous structure significantly enhanced CO_2_ adsorption capacity and regeneration performance. More recently, Wang *et al.*^18^ developed low-cost porous carbons from agricultural waste using a one-step alkali activation strategy and reported that the combined development of microporosity and oxygen- and nitrogen-containing functional groups effectively promoted CO_2_ capture. Although these studies demonstrate the effectiveness of one-step chemical activation for producing biomass-derived adsorbents, they primarily investigate a single activating agent or biomass precursor. Consequently, a systematic comparison of different activating agents under identical preparation conditions and their influence on pore development, surface chemistry, adsorption thermodynamics, CO_2_/N_2_ selectivity, and cyclic stability remains limited, particularly for Moroccan Alfa fiber-derived biochars. Inspired by these studies, the present work explores the enhancement of CO_2_ adsorption by Moroccan Alfa-derived biochar through a one-step chemical activation strategy, in which carbonization and activation are carried out simultaneously under an inert atmosphere.

Despite contributing little to global CO_2_ emissions, Morocco is proactively implementing measures to reduce its carbon footprint through renewable energy programs through its National Low Carbon Strategy. The nation has committed to achieving carbon neutrality by 2050, with a strong focus on renewable energy sources, including solar, wind, and hydropower.^19^ This investigation focuses on the preparation of activated biochar from Alfa fiber (Af) biomass, selected for its economic viability and widespread availability across Morocco and North Africa. The activation process employed potassium hydroxide (KOH), melamine, and zinc chloride (ZnCl_2_), each serving a distinct function to enhance the structural and surface properties of the resulting activated biochar and improve its CO_2_ capture performance. KOH and ZnCl_2_ primarily facilitate pore development, increasing surface area and promoting the formation of micro- and mesoporous architectures, which are crucial for gas adsorption and for achieving economically viable storage capacity for DAC applications.^20^ However, N_2_ is present in air at a substantially higher concentration than CO_2_; selective CO_2_ adsorption in the presence of N_2_ represents a fundamental challenge in adsorption-based gas separation. It is therefore essential to evaluate the CO_2_/N_2_ selectivity of Af-K1, a parameter that has been the subject of prior investigation in the literature^11^ and which represents an important criterion for evaluating the practical applicability of any candidate adsorbent material in CO_2_ capture processes.^21^ Conversely, melamine incorporates surface nitrogen functionalities that provide Lewis basic adsorption sites, potentially enhancing the biochar's affinity for CO_2_ molecules.^22^ Accordingly, the present study systematically compares three chemically distinct activating agents (KOH, ZnCl_2_, and melamine) under identical one-step activation conditions to elucidate their respective effects on pore development, surface chemistry, CO_2_ adsorption capacity, CO_2_/N_2_ selectivity, adsorption thermodynamics, and cyclic stability. By establishing the relationship between activation chemistry and adsorption performance, this work provides guidance for the rational design of biomass-derived adsorbents for CO_2_ capture applications (Table 1).

**Table 1:** Materials and chemicals used in the activation process

Chemical name	Purity	Supplier	Purpose
Alfa fiber (Af) (*Stipa tenacissima*)	Raw in natural form	Eastern Morocco	Use as biomass
Water (H_2_O)	Deionized grade pure	In-house supply from the tap	Use for washing
Nitrogen (N_2_)	≥99.99%	Linde	Inert gas
Hydrochloric acid (HCl)	37%	Merck	Use for washing
Potassium hydroxide (KOH)	≥85%	Sigma-Aldrich	Increase porosity
Zinc chloride (ZnCl_2_)	≥98%	Sigma-Aldrich	Increase porosity
Melamine (C_3_H_6_N_6_)	99%	Sigma-Aldrich	N-doped biochar

## 2. Experimental details

### 2.1. Biochar feedstock collection

Alfa fiber (Af), obtained from *Stipa tenacissima*, is considered one of the most abundant lignocellulosic resources in the Mediterranean region. Its composition primarily comprises cellulose (approximately 40–48%), hemicellulose (approximately 22–27%), and lignin (approximately 12–18%).^23^ This perennial grass grows in dry and semi-dry zones, particularly across North Africa. In Morocco, the Af spans an estimated area of 3.2 million hectares, with neighboring Algeria and Tunisia hosting nearly 4 million and 0.4 million hectares, respectively.^24^ In this study, Af were sourced from the Oriental region in eastern Morocco, located at coordinates 34.375348, −2.913861.

### 2.2. One-step Af-biochar activation

**Fig. 1:**
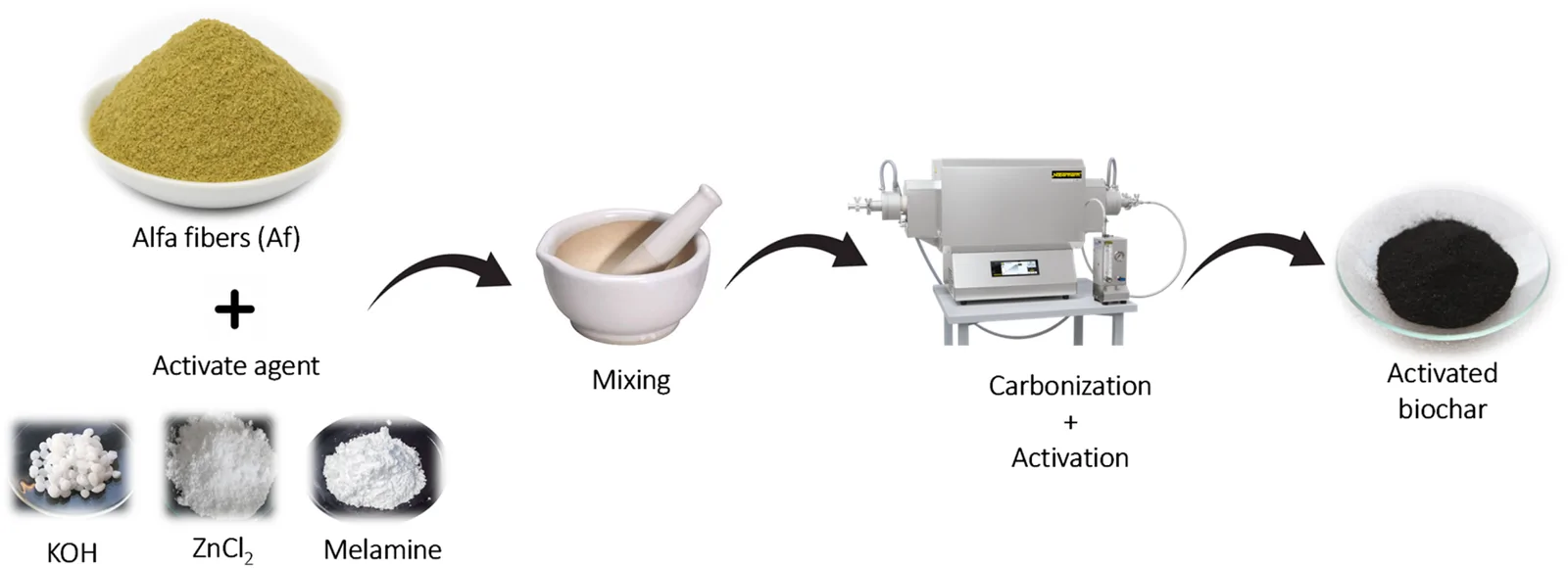
Schematic representation of activated biochar preparation from Alfa fibers.

After collection, Alfa fibers (Af) were rinsed thoroughly with deionized water to remove surface contaminants, then drained at ambient temperature. The cleaned fibers were subsequently oven-dried overnight at 100 °C. Once dried, the dried fibers were milled and sieved to produce fine biomass particles having an average particle size of 100 µm. A 1 g portion of Af was then homogeneously mixed with a measured quantity of activating agents, potassium hydroxide (KOH), melamine, or zinc chloride (ZnCl_2_), at various mass ratios of 0.5, 1.0, and 1.5 relative to the Alfa fiber. The mixtures were thoroughly homogenized in a mortar for 20 minutes to achieve homogeneous dispersion of the activating agents. The resulting blends underwent simultaneous carbonization and chemical activation by heating at 700 °C for 1 hour under a continuous N_2_ atmosphere to maintain inert conditions. Following thermal treatment, the activated biochars were washed with a 1 wt% HCl solution to remove most residual inorganic species, then rinsed repeatedly with deionized water until a neutral pH was reached. The resulting materials were finally dried overnight at 110 °C in a laboratory oven. All preparation steps are illustrated in Fig. 1. The prepared activated biochars were denoted as Af-X*y*, where “X” represents each activating agent (K for KOH, M for melamine, Zn for ZnCl_2_), and “*y*” denotes the mass ratio (0.5, 1.0, or 1.5). A reference sample, labeled Af-700, was prepared under the same thermal conditions but without any activating agent.

### 2.3. Characterization techniques

Proximate analysis (moisture, volatile matter, fixed carbon, and ash) was performed by thermogravimetric analysis (NETZSCH TG 209 F1 Libra). Approximately 4 mg of sample was heated from 25 to 900 °C at 20 °C min^−1^, with an isothermal hold at 105 °C for 7 min to determine moisture content, followed by 15 min of devolatilization under a nitrogen atmosphere to determine volatile matter, and 15 min of combustion under air at 900 °C to determine the fixed carbon and ash contents.

Elemental composition (CHNS) of the activated biochar was determined using a Thermo Flash 2000 elemental analyzer to determine the CHN-S content. The oxygen content was determined by difference, as commonly applied in elemental analysis,^16^ where the measured carbon (C), hydrogen (H), nitrogen (N), and sulfur (S) contents were subtracted from 100% according to: O (wt%) = 100 − (C + H + N + S + Ash).

FTIR analysis was performed on the samples using a PerkinElmer instrument. Before analysis, all samples were finely ground into powders (<63 µm particle size) to ensure uniformity and improve spectral accuracy.^25^ All spectra were recorded in transmittance mode within the wavenumber range of 4000–600 cm^−1^ using a spectral resolution of 4 cm^−1^ and 16 accumulated scans per sample.

X-ray diffraction (XRD) was employed to investigate the crystalline structure of the biochar using a D2 PHASER diffractometer (Bruker). Each sample was ground into powder and securely positioned on the sample holder to ensure consistent X-ray exposure. Sample preparation included the back-loading technique to minimize preferred-orientation effects. Scanning was conducted using CuK_α_ radiation (*λ* = 1.54056 Å) within the 2*θ* range of 15–80° with a scan rate of 0.01° s^−1^ and a step size of 0.02°. Generator settings were maintained at 30 kV and 10 mA to ensure optimal signal intensity while preventing sample degradation.

Raman spectroscopy was employed to investigate the molecular structure and bonding characteristics of the samples, complementing other analytical techniques. High-resolution Raman scattering spectra were acquired at room temperature using a HORIBA LABRAM-HR Evolution spectrometer equipped with a 532 nm laser operating at 1 mW to minimize sample heating. The laser spot diameter was approximately 1 µm using a 50× objective lens. Raman spectra were collected over the 500–3000 cm^−1^ spectral range at a resolution of 1 cm^−1^ and an acquisition time of 10 s per spectrum. Spectral deconvolution was performed using Lorentzian–Gaussian fitting to separate overlapping D and G bands.^26^ The D/G band area ratio (*A*_D_/*A*_G_), corresponding to the D band (∼1350 cm^−1^) and G band (∼1580 cm^−1^), was calculated to assess the degree of graphitization, providing information on the evolution of aromatic domains and the degree of carbon crystallite ordering. The *A*_D_/*A*_G_ ratio correlates inversely with crystallite size according to the Tuinstra–Koenig relationship: *L*_a_ (nm) = 4.4(*A*_D_/*A*_G_)^−1^.^27^ Additionally, changes in this ratio across different activation conditions reflected the development of aromatic ring structures and variations in crystallite size, providing insights into how activation agents influence carbon ordering.

X-ray photoelectron spectroscopy (XPS) measurements were carried out using a Kratos AXIS Ultra DLD spectrometer equipped with a monochromatic Al Kα X-ray source (1486.6 eV) operating at 150 W (10 mA, 15 kV). Spectra were acquired over an analysis area of approximately 700 × 300 µm^2^. High-resolution spectra were recorded using a 50 meV step size, a dwell time of 500–1000 ms, and a pass energy of 40 eV, and were averaged over several scans to enhance the signal-to-noise ratio. The Kratos charge-neutralizer was used to insulate samples. Binding energy values were calibrated by assigning the adventitious carbon C 1s peak to 284.8 eV. Samples were mounted on conductive adhesive tape on the sample holder prior to analysis.

The specific surface area was determined using the Brunauer–Emmett–Teller (BET) method, whereas the pore size distribution was evaluated using the Barrett–Joyner–Halenda (BJH) method based on N_2_ adsorption–desorption isotherms acquired with a Micromeritics TRISTAR II PLUS analyzer. Prior to analysis, the samples were degassed under vacuum at 120 °C for 12 h to eliminate moisture and contaminants, ensuring accurate and reproducible results.

Surface morphology was examined by scanning electron microscopy (SEM) using a field-emission scanning electron microscope (FE-SEM, Zeiss Evo 10) operated at an accelerating voltage of 15 kV. Prior to imaging, the samples were carefully mounted on carbon adhesive disks, followed by sputter coating with a thin layer of gold for approximately 5 minutes at a supply current of 20 mA. This step was essential for improving imaging quality and minimizing surface charging under the electron beam. SEM imaging was used to analyze surface irregularities and pore formation, and the results were visually assessed to understand the effects of different activation agents. Elemental composition and spatial distribution were determined by energy-dispersive X-ray spectroscopy (EDX) using an Oxford Instruments detector with AZtec (Tru-Q) software, with Map Sum Spectrum acquisition over the imaged area.

Transmission electron microscopy (TEM) was performed to investigate particle morphology and structural features. Approximately 30 mg of finely ground powder was dispersed in ethanol and ultrasonicated for 20 min. Three drops of the prepared suspension were placed onto a carbon-coated copper grid using a micropipette. High-resolution TEM (HRTEM) imaging was conducted using a Thermo Fisher Talos F200i TEM operated at 200 kV. Furthermore, elemental distribution was characterized by scanning transmission electron microscopy (STEM) combined with energy-dispersive X-ray spectroscopy. High-angle annular dark-field (HAADF) imaging was employed to acquire STEM-EDX elemental maps, providing nanoscale compositional contrast.

### 2.4. CO_2_ adsorption measurement

The CO_2_ adsorption capacity of the activated biochar at RH = 0 and 30 °C over a pressure range of 0–1 bar was determined using a Gaspro GA apparatus from SETARAM. Approximately 0.1 g (±0.001 g) of activated biochar was introduced into a microdoser for each experiment. Prior to testing, the samples were degassed under vacuum (10^−6^ bar) at 120 °C for 12 h to eliminate residual moisture and impurities. CO_2_ adsorption measurements commenced once the temperature stabilized at 30 °C, ensuring equilibrium before data collection. Nitrogen (N_2_) adsorption isotherms were also measured on the same apparatus under identical conditions (30 °C and 0–1 bar) to evaluate CO_2_/N_2_ selectivity. The tests were conducted in accordance with standardized protocols to ensure a consistent and accurate assessment of the activated biochar's adsorption performance. Cyclic adsorption–desorption was conducted as follows: after reaching adsorption equilibrium at 30 °C and 1 bar, the system automatically evacuated the sample tube to a pressure below 0.01 bar to enable CO_2_ desorption. The adsorption step was then repeated under the same conditions. This adsorption–desorption procedure was performed ten consecutive times to assess the cyclic CO_2_ capture performance of the Af-K1 sample.

### 2.5. Adsorption isotherms models

The Freundlich isotherm^28^ is an empirical model describing adsorption on heterogeneous surfaces with sites of different adsorption energies.^29^

1 *q* = *k*_F_*P*^1/*n*^

The Langmuir model assumes monolayer adsorption, meaning that each adsorption site can accommodate only one adsorbate molecule, and interactions between neighboring adsorbed molecules are neglected.^30^2
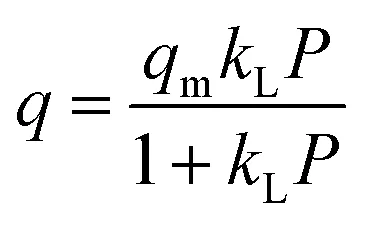
where *q* (mmol g^−1^) is the equilibrium adsorption capacity, *q*_m_ (mmol g^−1^) is the maximum adsorption capacity, *P* (bar) is the equilibrium pressure, *k*_F_ (mmol g^−1^ bar^−1/*n*^), and *k*_L_ (bar^−1^) are the Freundlich and Langmuir constants, and *n* is the heterogeneity parameter.

The Redlich–Peterson isotherm is a three-parameter model combining the characteristics of the Langmuir and the Freundlich isotherms, making it suitable for describing both homogeneous and heterogeneous adsorption.^16^ Its nonlinear form is given by eqn (3):^31^3
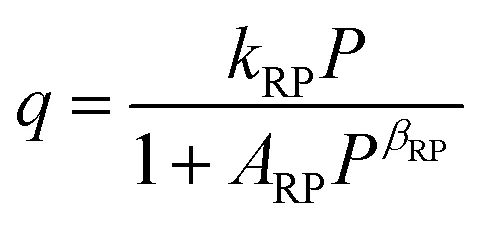
where *k*_RP_ (mmol g^−1^ bar^−1^) and *A*_RP_ (bar^−*β*_RP_^) are isotherm constants, and *β*_RP_ is the model exponent (0 < *β*_RP_ ≤ 1). The value of *β*_RP_ indicates the deviation from ideal Langmuir behavior.

The Sips model was also applied to describe the adsorption equilibrium.^32^ The equation is expressed as:4
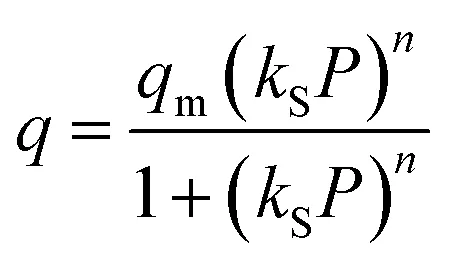
where *q* (mmol g^−1^) is the equilibrium adsorption capacity, *q*_m_ (mmol g^−1^) is the maximum capacity, *k*_S_ (bar^−1^) is the Sips affinity constant, *P* (bar) is the equilibrium pressure, and *n* is the dimensionless heterogeneity parameter.

### 2.6. CO_2_ adsorption thermodynamic analysis

Gibbs free energy (Δ*G*°) is a thermodynamic parameter used to evaluate the spontaneity of an adsorption process. Δ*G*° was determined at various temperatures using the standard relation.^33^

5 Δ*G*° = −*RT* ln(*K*°)

where *R* is the universal gas constant, *T* is the absolute temperature (K), and *K*° is the dimensionless equilibrium constant obtained from the pressure-based Langmuir constant (*K*° = *K*_L_ × *P*°, *P*° = 1 bar).^34,35^

The standard enthalpy (Δ*H*°) (kJ mol^−1^) and entropy (Δ*S*°) (kJ mol^−1^ K^−1^) were determined from the slope and intercept of the Van't Hoff plot of ln(*K*°) *versus* 1/*T* (Fig. S6), respectively:^36^

6 Δ*G*° = Δ*H* − *T*Δ*S*°

7
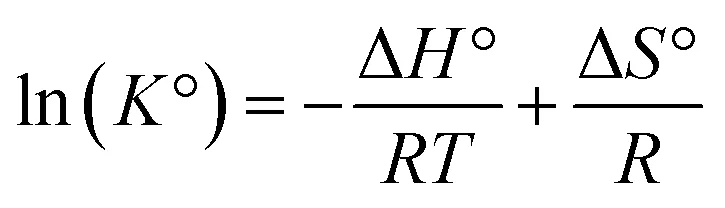


The thermodynamic parameters were obtained from equilibrium adsorption data measured at different temperatures using dimensionless equilibrium constants derived from the Langmuir isotherm to ensure thermodynamic consistency in the calculations.

The isosteric heat of adsorption 
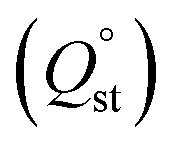
 was determined to evaluate the interaction strength between CO_2_ molecules and the adsorbent surface. 
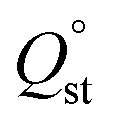
 was determined from adsorption isotherms collected at 0, 30, and 45 °C using the Clausius–Clapeyron equation.^37^8
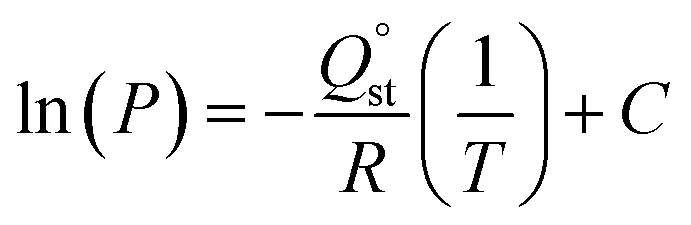
where 
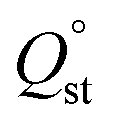
 is expressed in kJ mol^−1^, *P* is the equilibrium pressure (bar), *R* is the universal gas constant, *T* is the absolute temperature (K), and *C* is a constant.

The equilibrium pressures corresponding to each adsorption loading were obtained from the experimental isotherms measured at the three temperatures. The isosteric heat of adsorption was obtained from the slope of the ln(*P*) *versus* 1/*T* plot following the procedure reported by J. Singh *et al.*^38^ Accordingly, 
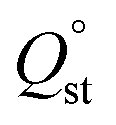
 was calculated as9
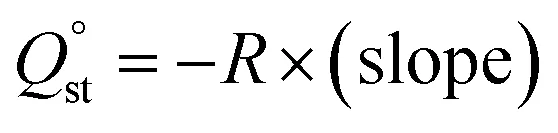


## 3. Results and discussion

### 3.1. Physicochemical characterization of the activated Af-biochar

The biochar yields ranged from 19.7 to 29.3 wt% (Table S1) and were strongly dependent on the nature of the activating agent. The melamine-modified samples showed the highest yields, increasing from 25.9 to 29.3 wt% as the melamine ratio increased, exceeding those of the non-activated biochar (Af-700, 24.2 wt%). This is attributed to melamine acting as a nitrogen source that deposits nitrogen-rich carbonaceous residue onto the char rather than etching the carbon framework, consistent with the pronounced rise in nitrogen content determined by CHNS elemental analysis (from 4.47 to 15.43 wt% for Af-M0.5 and Af-M1.5, respectively). In contrast, the ZnCl_2_- and KOH-activated samples exhibited lower yields that decreased with increasing impregnation ratio (22.2–20.4 wt% for ZnCl_2_ and 20.5–19.7 wt% for KOH), consistent with carbon gasification and framework etching, which are responsible for pore development. The proximate analysis of the Alfa fiber-derived biochars (Table S1) provides insight into their bulk composition, including moisture, volatile matter, fixed carbon, and ash. All samples exhibited high fixed-carbon contents (60–79 wt%), confirming their highly carbonaceous nature after thermal treatment. The non-activated biochar (Af-700) showed the highest fixed carbon (78.52 wt%) and a moderate ash content (6.66 wt%), the latter reflecting the mineral matter naturally present in the Alfa fiber. Chemical activation altered the ash content in an agent-dependent manner: the KOH-activated samples exhibited the lowest ash contents (2.27–3.98 wt%), consistent with the dissolution of silica and other mineral species by KOH and their subsequent removal during acid washing, whereas the ZnCl_2_-activated samples retained higher ash contents (3.49–5.86 wt%). The melamine-modified sample Af-M1.5 showed the highest ash content (10.97 wt%), indicating a greater residual inorganic fraction. The relatively high moisture contents of some activated samples (up to ∼20 wt%) reflect their hygroscopic character, arising from the developed porosity and the abundance of surface functional groups. The measured ash contents were used to calculate the oxygen values reported by difference, providing a more reliable estimate of the elemental composition. Ultimate analysis of the Alfa fiber-derived biochars (Table S1) shows clear variations in surface chemistry depending on the activation agent and its ratio. The reference sample Af-700 contains moderate carbon (48.68 wt%) and the highest oxygen content (42.05 wt%), indicating limited deoxygenation during pyrolysis alone. In contrast, the KOH-activated samples (Af-K0.5, Af-K1, Af-K1.5) exhibit higher carbon content (57–63 wt%) and lower oxygen content (31–39 wt%), consistent with the strong deoxygenating and gasifying action of KOH. The melamine-treated samples show a distinct trend: the nitrogen content rises markedly from 4.47 wt% to 15.43 wt% with increasing melamine ratio, confirming efficient nitrogen doping, accompanied by a reduction in oxygen content as nitrogen functionalities are incorporated during pyrolysis. The ZnCl_2_-activated biochars exhibit intermediate carbon contents (52–61 wt%) and relatively high oxygen contents (31–37 wt%), reflecting the dehydrating and pore-forming behavior of ZnCl_2_, which promotes microporosity while retaining oxygenated structures. Overall, these results indicate that KOH enhances the carbon content through aggressive gasification, melamine enriches the surface with nitrogen functionalities that may strengthen CO_2_ interactions, and ZnCl_2_ preserves oxygen-rich networks that contribute to physisorption. These compositional differences influence the surface chemistry and, ultimately, the CO_2_ adsorption performance of the resulting biochars.

Fig. S1a displays the FTIR spectra of Af-700 and Af-derived activated biochar with KOH, Melamine, and ZnCl_2_ at 700 °C. All samples display a peak in the range of 3000–3500 cm^−1^, which could indicate O–H or N–H stretching vibrations, common to hydroxyl or amine groups.^39^ The reference sample (Af-700) shows fewer, weaker peaks, characteristic of a simpler, non-functionalized carbon structure. In contrast, each activation agent introduces distinct features: KOH-treated samples exhibit significant changes in the 1500–2500 cm^−1^ region, likely reflecting the addition of oxygenated functional groups (C=C, C–O) and enhanced carbon structures.^40^ ZnCl_2_-activated samples show peaks in the 1000–700 cm^−1^ range, corresponding to the aromatic structure (Ar–H).^41^ Melamine-activated samples exhibit peaks near 1600–1500 cm^−1^ and 978–1210 cm^−1^, indicative of nitrogen-containing functional groups (C–N or N–H bonds), confirming nitrogen incorporation. Overall, the spectra highlight how different activation agents (KOH, ZnCl_2_, melamine) and their concentrations modify the chemical functionalities and structure of the samples. These modifications, such as the development of porosity, increased surface area, and the introduction of nitrogen-containing groups, create more accessible and chemically favorable sites for CO_2_ molecules, thereby enhancing the adsorption capacity.

Fig. S1b represents the XRD pattern of biochar prepared from Af activated in one step with KOH, ZnCl_2_, and melamine at 700 °C, which reveals significant structural and compositional changes influenced by the activation agents. A broad peak around 20°–30° and 40°–45° (2*θ*) corresponding to the (002) plane and (100), respectively, indicates the presence of amorphous carbon,^16^ with variations in intensity reflecting the degree of graphitization. KOH-activated biochar exhibits enhanced amorphous carbon characteristics with minimal residual crystalline phases, indicating that KOH primarily induces porosity and structural defects.^42^ In contrast, ZnCl_2_-activated samples showed a minor difference in the XRD pattern, which can be attributed to residual inorganic species retained after washing. Consistent with this, EDX (Fig. S3) and XPS analyses confirmed the presence of residual zinc, predominantly as ZnO (Zn 2p_3/2_ at ∼1023 eV), together with trace chlorine and minor mineral impurities originating from the Alfa fiber.^43^ Melamine activation does not produce crystalline nitrogen-containing phases but likely introduces nitrogen functionalities, such as pyridinic-N, pyrrolic-N, and graphitic-N, into the amorphous carbon matrix.^44^ These basic nitrogen groups can enhance CO_2_ adsorption by increasing the surface's affinity through acid–base interactions. Residual CaCO_3_ peaks,^45^ derived from natural impurities in the Af, are present in some samples, indicating incomplete removal during activation. Overall, the analysis demonstrates that KOH and ZnCl_2_ play critical roles in tailoring the porosity and promoting structural ordering within the carbon matrix. In contrast, melamine primarily facilitates nitrogen doping by introducing surface functional groups.

**Fig. 2:**
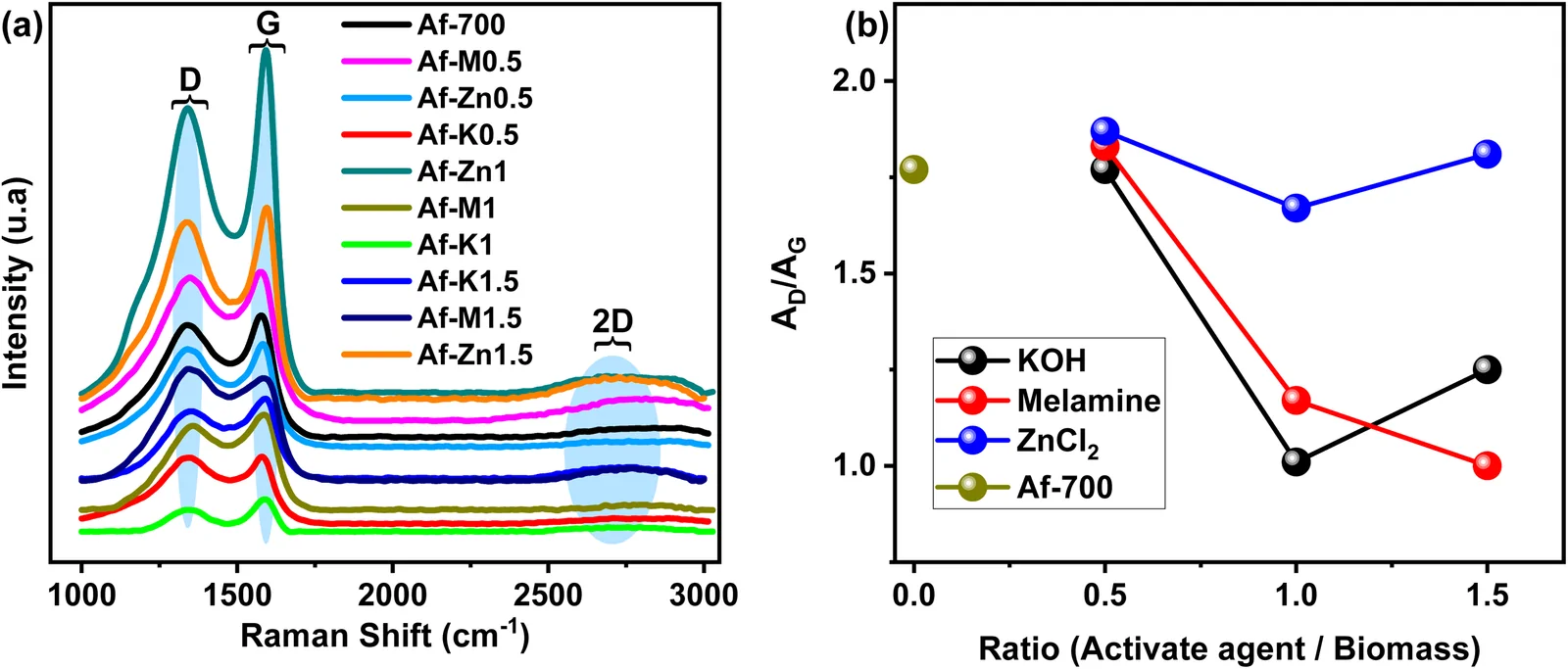
(a) Raman spectra and (b) *A*_D_/*A*_G_ band ratio of Af-derived activated biochar.

Fig. 2a displays the Raman spectra of all samples, highlighting distinct D-bands (1200–1450 cm^−1^), G-bands (1500–1700 cm^−1^), and 2D bands (2650–2750 cm^−1^). The D-band corresponds to structural disorder and defects associated with A_1g_ symmetry of sp^3^ carbon vibrations,^46^ while the G-band indicates a graphitic structure related to the E_2g_ phonon of sp^2^ carbon atoms.^47^ The 2D band offers insights into the organization of graphene-like layers and the extent of graphitization in these materials.^48^

The degree of disorder in each sample was assessed using the *A*_D_/*A*_G_ ratio (the area ratio of the D- and G-bands), as shown in Fig. 2b. These *A*_D_/*A*_G_ values highlight variations in structural disorder and graphitization, influencing their potential applications for CO_2_ capture based on defect density. Sample Af-700, with the highest ratio (1.73), exhibits significant structural disorder and is highly amorphous, enhancing its surface reactivity.^49^ Af-K0.5 and Af-K1, with *A*_D_/*A*_G_ ratios of 1.77 and 1.01, respectively, exhibit lower disorder than Af-700, suggesting a balance between amorphous and graphitic features suitable for moderate CO_2_ adsorption applications. Af-K1.5, has an *A*_D_/*A*_G_ ratio of 1.25, which falls within a similar range, maintaining a moderate level of structural defects and reactivity.^49^ Samples (Af-Zn0.5, Af-Zn1, and Af-Zn1.5) activated with ZnCl_2_ show *A*_D_/*A*_G_ values of 1.87, 1.67, and 1.81, respectively. Af-Zn1, with the lowest ratio, has a more graphitic and less defective structure, whereas Af-Zn0.5, with its higher ratio, reflects an increased defect density. Samples treated with melamine (Af-M0.5, Af-M1, and Af-M1.5) exhibited *A*_D_/*A*_G_ values of 1.83, 1.17, and 1.01, respectively, indicating a high degree of structural disorder. These samples, particularly Af-M1 and Af-M0.5, show potential for CO_2_ adsorption due to their highly defective surfaces, which can enhance gas adsorption. Importantly, the relative increase or decrease of the D and G bands compared to the reference Af-700 reflects the structural modifications induced by the activating agents: an increase in the D band intensity (higher *A*_D_/*A*_G_) corresponds to the creation of more defects, edge sites, and amorphous domains that provide active sites for CO_2_ binding, while an increase in the G band intensity (lower *A*_D_/*A*_G_) indicates the development of more ordered graphitic domains that enhance structural stability and conductivity.^50^ Therefore, the up-and-down variations in the D and G bands are critical, as they reveal the delicate balance between disorder and graphitization, both of which play decisive roles in determining the adsorption performance and stability of the prepared carbons.^26^

**Fig. 3:**
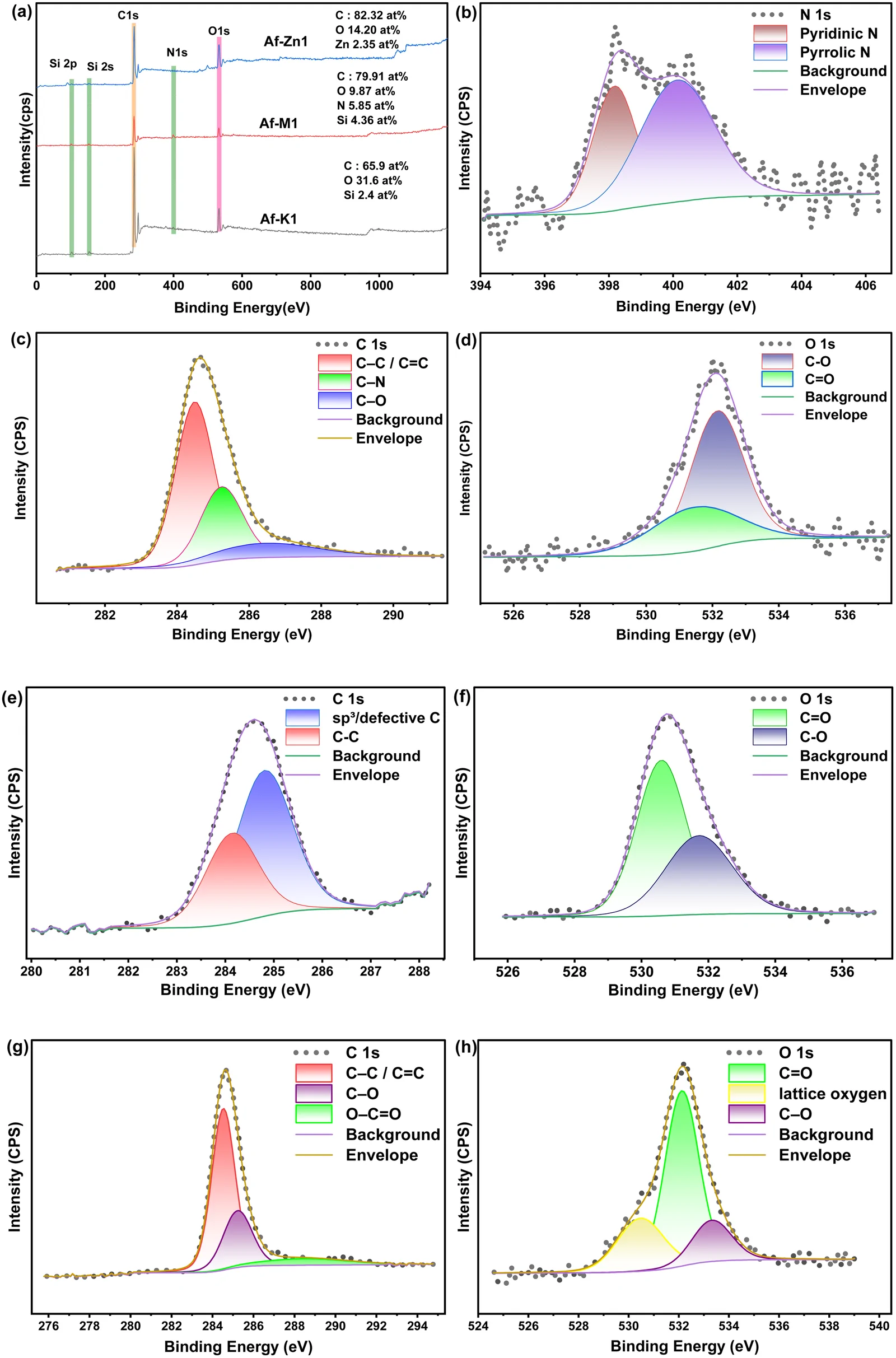
(a) Survey spectra; (b–d) high-resolution C 1s, O 1s, and N 1s spectra of Af-M1; (e and f) and (g and h) high-resolution C 1s and O 1s spectra of Af-K1 and Af-Zn1, respectively.

Fig. 3a shows the XPS survey spectra of Af-K1, Af-M1, and Af-Zn1, confirming carbon and oxygen as the principal surface elements, with nitrogen present in Af-M1, zinc in Af-Zn1, and trace silicon originating from biomass inorganics.^23^ The atomic surface compositions, normalized to 100%, were C 65.9, O 31.6, Si 2.4 at% for Af-K1; C 79.9, O 9.9, N 5.9, Si 4.4 at% for Af-M1; and C 82.3, O 14.2, Zn 2.4, Si 1.1 at% for Af-Zn1.

For the melamine-modified sample Af-M1, the N 1s spectrum (Fig. 3b) exhibited two components at 398.2 and 400.0 eV, corresponding to pyridinic and pyrrolic nitrogen, respectively, which act as Lewis-basic sites that favor CO_2_ adsorption.^40^ The C 1s spectrum (Fig. 3c) was deconvoluted into three components at 284.5, 285.3, and 286.3 eV, assigned to graphitic carbon (C–C/C=C), nitrogen-bonded carbon (C–N), and oxygenated carbon (C–O), respectively; the C–N contribution confirms the incorporation of nitrogen from melamine. The O 1s spectrum (Fig. 3d) was fitted with two components at 531.1 eV (C=O) and 532.2 eV (C–O).

For the KOH-activated sample Af-K1, the C 1s spectrum (Fig. 3e) was resolved into two components at 284.1 eV (graphitic C–C/C=C) and 284.8 eV (sp^3^/defective carbon), with no feature below 283 eV; the predominance of sp^3^/defective carbon is consistent with the strong etching action of KOH. The O 1s spectrum (Fig. 3f) showed two components at 530.7 eV (C=O) and 531.8 eV (C–O), in agreement with the high oxygen content (31.6 at%) from the survey.^51^

For the ZnCl_2_-activated sample Af-Zn1, the C 1s spectrum (Fig. 3g) was deconvoluted into three components at 284.6, 285.2, and 288.4 eV, attributed to graphitic carbon (C–C/C=C), oxygenated carbon (C–O), and carboxyl/ester carbon (O–C=O), respectively. The O 1s spectrum (Fig. 3h) was resolved into three components at 530.5, 532.1, and 533.3 eV, assigned to lattice/metal-oxide oxygen (Zn–O), carbonyl oxygen (C=O), and hydroxyl/ether oxygen (C–O), respectively. The lattice-oxygen component, together with the Zn 2p signal at ∼1023 eV, indicates residual ZnO retained after activation and washing. Overall, the XPS results show that melamine introduces nitrogen-containing basic sites, KOH produces an oxygen-rich, structurally disordered carbon surface, and ZnCl_2_ leaves residual ZnO alongside oxygen-rich functionalities.

**Fig. 4:**
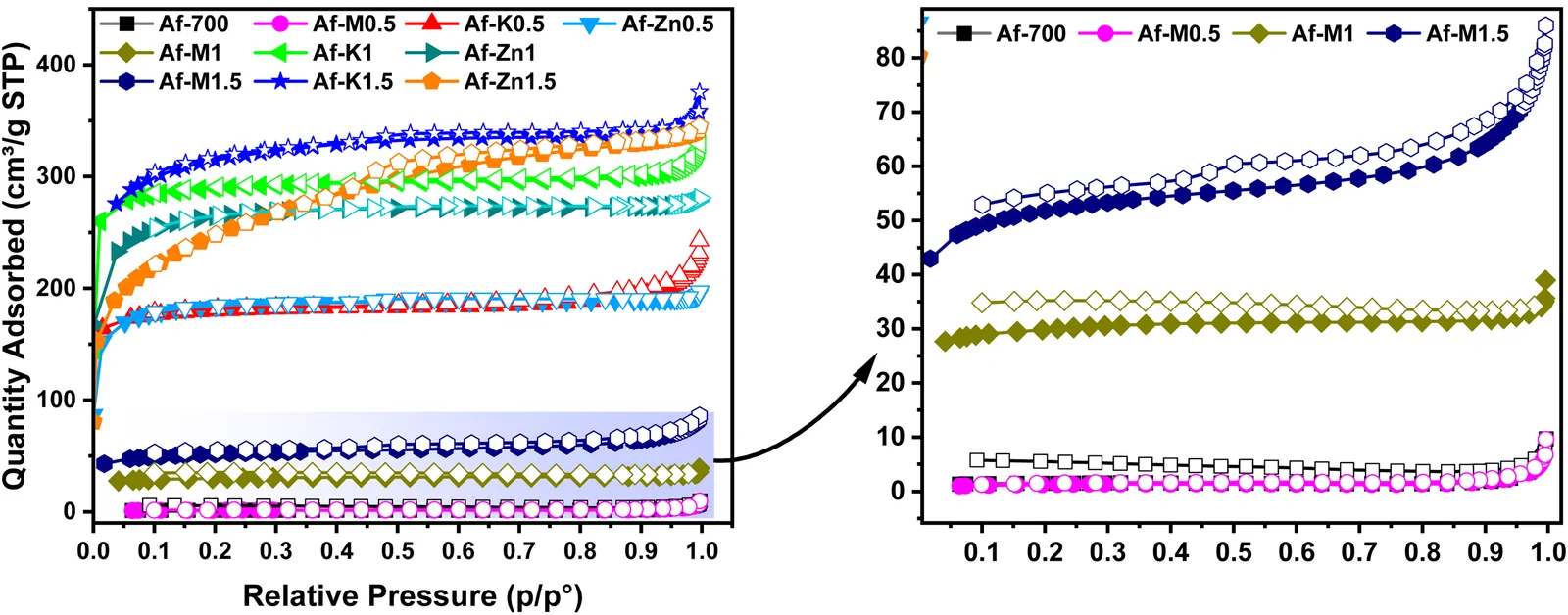
N_2_ adsorption (closed symbols) – desorption (open symbols) isotherms at −196 °C.

Fig. 4 presents the N_2_ adsorption–desorption isotherms of the prepared activated biochar samples. Based on the IUPAC classification, all activated biochars display combined Type I and Type IV isotherms,^52^ suggesting the existence of micro- and mesopores, respectively.^53^ The presence of hysteresis loops, specifically type 1 (H1) and type 3 (H3), suggests a combination of cylindrical pores and wedge-shaped or groove-like pores with openings at both ends.^54^ The textural parameters, including surface area, pore volume, and pore size, summarized in Table 2, highlight the effect of chemical activation on the textural characteristics of Af-derived biochar. The reference sample (Af-700), carbonized at 700 °C without an activating agent, possesses a low specific surface area of 6 m^2^ g^−1^ and a total pore volume of 0.015 cm^3^ g^−1^, with mesopores contributing the majority (0.014 cm^3^ g^−1^) and an average pore size of 33.24 nm. This limited porosity reflects incomplete removal of volatiles and the absence of pore-forming activation agents.^55^ KOH activation produced the highest specific surface areas among the prepared samples, with Af-K1 exhibiting 1148 m^2^ g^−1^ and Af-K1.5 attaining a maximum of 1203 m^2^ g^−1^. These samples exhibit a well-developed porous structure, characterized by high micropore contributions (923 and 762 m^2^ g^−1^, respectively) and a considerable fraction of mesopores, making them particularly effective for adsorption applications. The pore sizes of KOH-activated samples decreased from 6.42 to 2.66 nm as the activation ratio increased from 0.5 to 1.5. This decrease indicates that higher KOH content promotes the development of narrower pores, particularly micropores, which enhance CO_2_ adsorption through stronger interactions with gas molecules. In contrast, ZnCl_2_ activation promotes microporosity. Af-Zn0.5 exhibits a surface area of 706 m^2^ g^−1^, with micropores contributing 447 m^2^ g^−1^ and a micropore volume of 0.150 cm^3^ g^−1^. As the ZnCl_2_ ratio increases (Af-Zn1 and Af-Zn1.5), the surface area reaches 1016 and 902 m^2^ g^−1^, respectively, with a pore size distribution favoring smaller micropores (1.87–2.53 nm). The decrease at the highest ratio indicates excessive activation, leading to pore collapse.^56^ Activation with melamine (Af-M0.5 and Af-M1) results in a moderate increase in surface area, reaching 112 m^2^ g^−1^ at a 1 : 1 ratio, where micropores account for 86 m^2^ g^−1^. Further increasing the melamine ratio to 1.5 enhances both surface area and total pore volume (197 m^2^ g^−1^ and 0.133 cm^3^ g^−1^, respectively), yielding a more balanced micro- and mesoporous structure. The resulting hierarchical pore structures, comprising both micropores and mesopores, are advantageous for gas adsorption applications, including CO_2_ capture, hydrogen,^55^ and methane^57^ storage. Pore size distribution (Fig. S2) indicates that ZnCl_2_-modified samples are microporous, whereas KOH-modified samples exhibit a more balanced distribution of micro- and mesopores, with fewer macropores.

**Table 2:** Textural characteristics of the Af-derived activated biochars, including BET surface area (*S*_BET_), micropore surface area (*S*_micro_), total pore volume (*V*_tot_), micropore volume (*V*_micro_), mesopore volume (*V*_meso_), average pore diameter, and CO_2_ adsorption capacity measured at 30 °C and 1 bar

Samples	*S*_BET_ (m^2^ g^−1^)	*S*_micro_ a (m^2^ g^−1^)	*V*_tot_ (cm^3^ g^−1^)	*V*_micro_ a (cm^3^ g^−1^)	*V*_meso_ b (cm^3^ g^−1^)	Pore size^c^ (nm)	CO_2_ uptake (mmol g^−1^)
Af-700	6	3	0.015	0.001	0.014	33.24	1.67
Af-M0.5	5	2	0.015	0.001	0.014	8.83	1.49
Af-Zn0.5	706	447	0.305	0.150	0.155	1.72	1.60
Af-K0.5	705	575	0.375	0.211	0.164	6.42	2.06
Af-M1	112	86	0.060	0.032	0.028	4.28	1.90
Af-Zn1	1016	654	0.434	0.216	0.218	1.87	2.33
Af-K1	1148	923	0.509	0.332	0.177	4.99	3.70
Af-M1.5	197	124	0.133	0.041	0.092	7.15	1.42
Af-Zn1.5	902	379	0.533	0.087	0.446	2.53	1.32
Af-K1.5	1203	762	0.581	0.251	0.330	2.66	3.01

a Micropore surface area and micropore volume determined by the *t*-plot method.

b By difference (*V*_tot_ − *V*_micro_ = *V*_meso_).

c Pore diameter by BJH adsorption (4*V*/*A*).

**Fig. 5:**
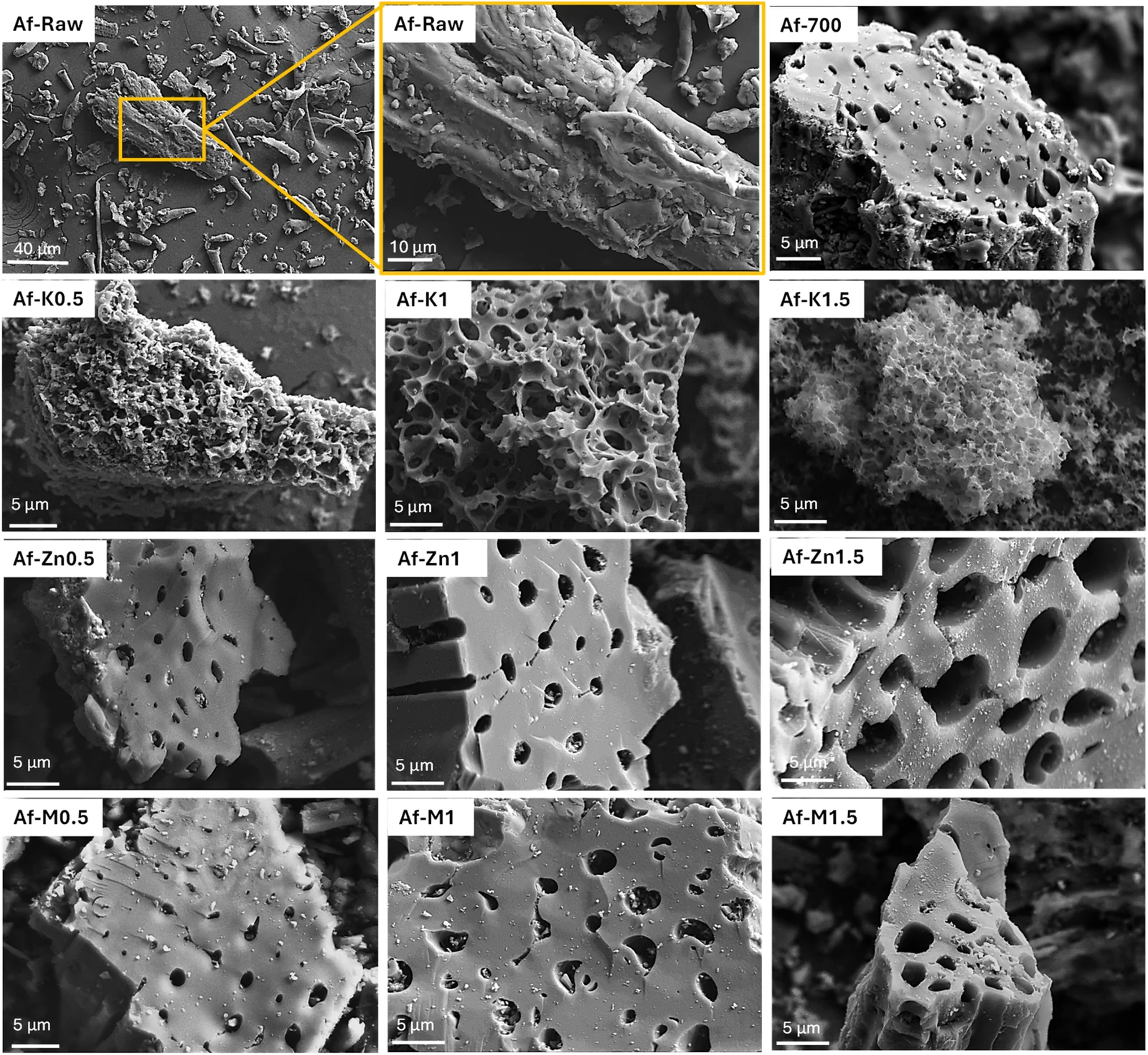
SEM images of Af biomass and their derived activated biochar.

Fig. 5 shows the morphological changes in Af and Af-activated biochar samples as observed by scanning electron microscopy (SEM), revealing significant transformations after pyrolysis and chemical activation with different agents and ratios. Originally, Af (Af-raw) looks dense, compact, and fibrous, like a typical raw biomass composed of cellulose, hemicellulose, and lignin. The Af (Af-700) sample exhibited the development of pores on its surface, attributed to thermal activation at 700 °C. This process created initial porosity by decomposing biomass and releasing volatile components, such as biopolymers and gases including CO_2_, H_2_, CH_4_, and CO. KOH produced Af (Af-K0.5/Af-K1/Af-K1.5), a highly porous, sponge-like, interconnected network with micro-, meso-, and macropores. It has been suggested that the alkaline mass ratio increases porosity during KOH activation, indicating that multiple mechanisms work at once: the intercalation of metallic potassium between graphitic layers, which expands the carbon structure and creates more porosity; the chemical etching of the carbon framework by potassium compounds; and the *in situ* formation of H_2_O and CO_2_, which react with carbon and contribute to pore development through a physical activation pathway.^58^ ZnCl_2_-treated activated carbon promotes template-like pore formation, resulting in more uniform honeycomb-like channels, as demonstrated by Zhang *et al.*^59^ when activating cork dust using ZnCl_2_. At the lowest mass ratio, the Af exhibited a relatively smooth surface with limited porosity, suggesting minimal activation. However, increasing ZnCl_2_ concentrations increased the number of pores. When the ZnCl_2_ ratio was increased to 1.5, larger pores formed, but irregularities and pore collapse were observed, indicating that high ZnCl_2_ concentrations may compromise structural integrity.^60^ Alternatively, the nitrogen-rich compound melamine was used to activate the surface of Af at different mass ratios (Af-M0.5, Af-M1, Af-M1.5), resulting in a honeycomb-like structure similar to that observed in ZnCl_2_-activated biochar, but with thicker walls. The sample Af-M0.5 remained dense with few pores, while the sample Af-M1 showed some pore development, mainly mesopores. At an even higher melamine ratio (Af-M1.5), the porosity increased with larger pores, although it remained less porous than the KOH- or ZnCl_2_-treated samples.

Considering all the effects, the most effective activating agent was KOH, which, through aggressive chemical activation and gasification of carbon, produced highly microporous, sponge-like carbon structures with interconnected micro–mesopores. This morphology, consistently reported to enhance CO_2_ adsorption, arises from the formation of micropores (<2 nm) critical for strong CO_2_ binding at ambient pressures, while mesopores facilitate rapid gas diffusion and effective utilization of the internal surface area. Previous studies^61,62^ have confirmed that such hierarchical porosity and high defect density created by KOH not only maximizes surface area but also provides abundant adsorption sites, resulting in CO_2_ uptakes as high as 8–9 mmol g^−1^ in comparable biomass-derived carbons.^63^ In contrast, ZnCl_2_ primarily generates micropores *via* template-directed mechanisms and exhibits moderate activation efficiency, whereas melamine functions primarily as a chemical modifier, introducing nitrogen functionalities without significantly enhancing porosity. Overall, these findings highlight the superior activation capabilities of KOH, with melamine and ZnCl_2_ serving complementary roles in tailoring the chemical and structural properties of Af-derived biochar. SEM-EDX analysis of the ZnCl_2_-activated samples (Af-Zn0.5, Af-Zn1, Af-Zn1.5; Fig. S3) confirmed carbon and oxygen as the dominant elements, along with residual zinc (3.0–3.9 wt%) and trace chlorine (0.6–0.9 wt%). This indicates that the HCl washing was reduced but did not fully remove the zinc species. The elemental maps show zinc uniformly distributed across the carbon matrix, consistent with the XPS results identifying residual ZnO (Zn 2p_3/2_ at ∼1023 eV).

**Fig. 6:**
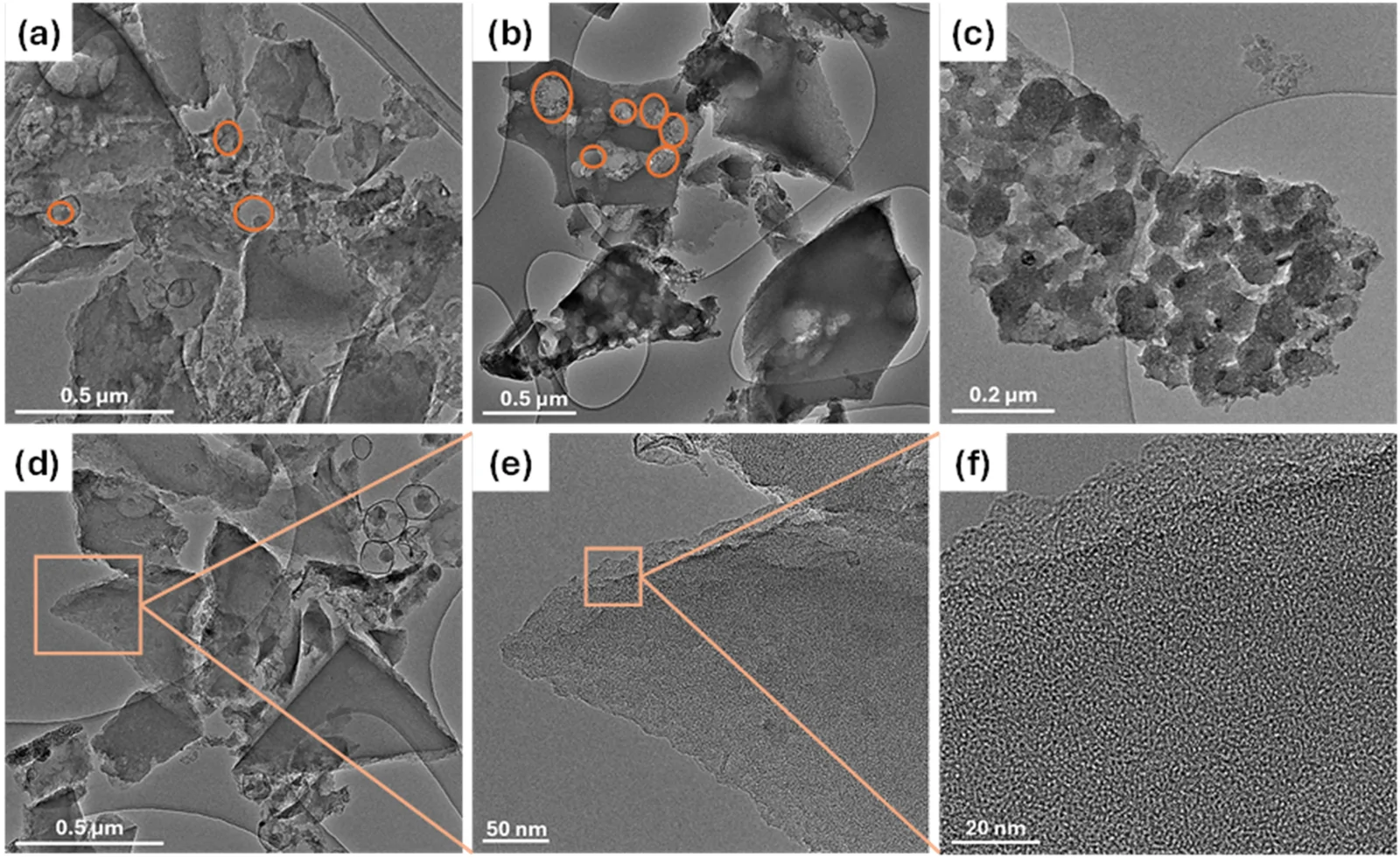
Bright field TEM micrographs of different morphologies for the Af-K1 particles, (a–d). HR-TEM micrographs (e and f).

The TEM micrographs of the Af-K1 sample (Fig. 6) demonstrate the development of a hierarchical porous architecture resulting from KOH activation of Af. The image in (a) reveals an irregular, expanded carbon framework containing numerous pore openings formed by the chemical etching action of KOH during activation. Fig. 6b exhibits a sponge-like morphology with interconnected micro- and mesopores, suggesting effluxion gas during the pyrolysis process. In Fig. 6c, regions of heterogeneous contrast are visible, indicating the coexistence of amorphous and semi-ordered carbon domains, suggesting partial graphitization of the carbon matrix. This observation is consistent with the Raman spectroscopy results, in which an *A*_D_/*A*_G_ intensity ratio of 1.01 confirms the presence of both disordered and graphitic structures in the material. The HR-TEM images (Fig. 6d–f) show a highly disordered carbon microstructure without clearly resolved lattice fringes, indicating the absence of long-range graphitic ordering. This confirms that the biochars remain predominantly amorphous at the nanoscale, with structural disorder dominating the carbon framework.

**Fig. 7:**
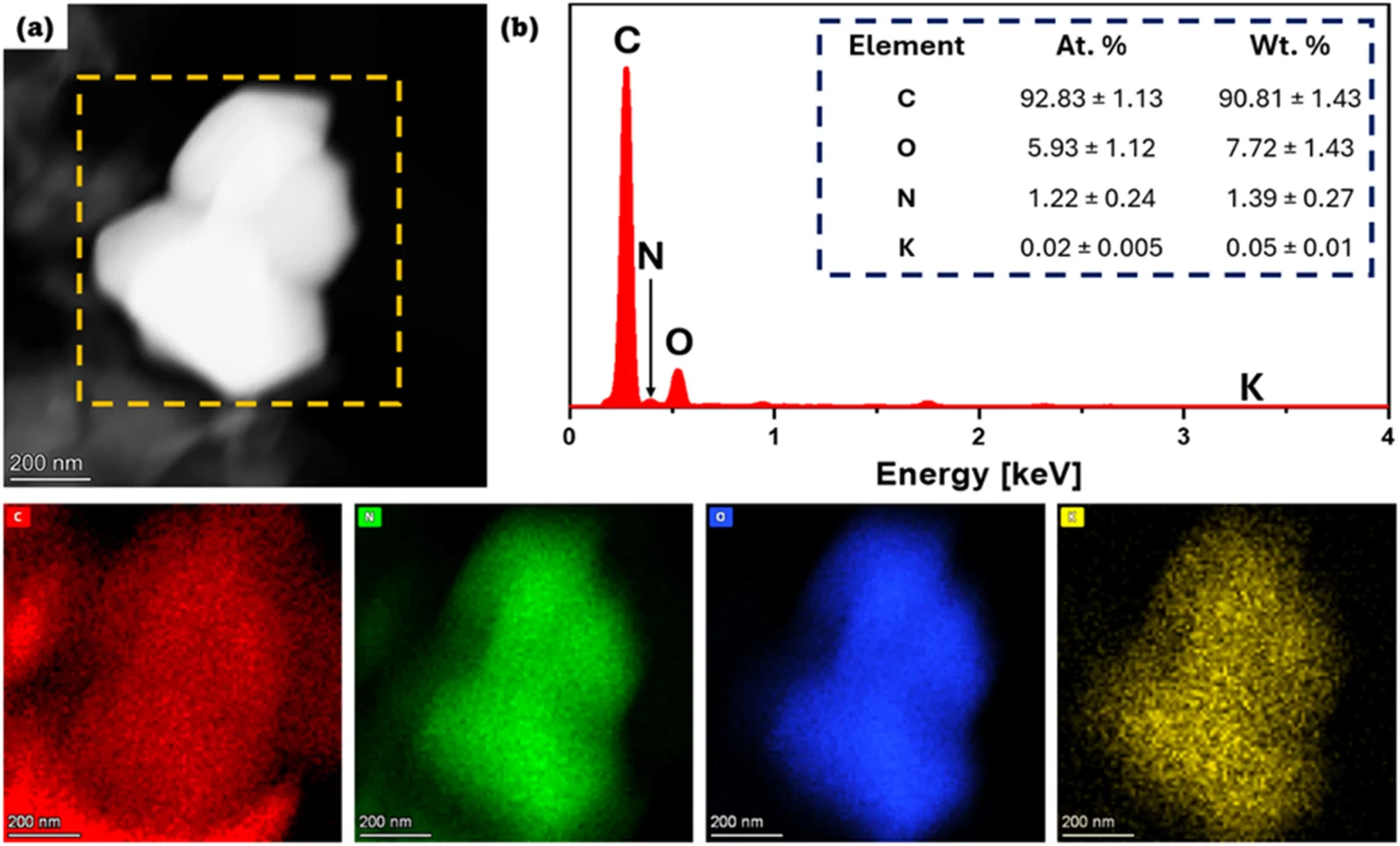
(a) HAADF-STEM image of an Af-K1 sample's particle, (b) EDX spectrum of the plotted area with corresponding elemental values of elements and EDX mapping.

EDX elemental mapping of the KOH-activated biochar Af-K1 (Fig. 7) reveals a uniform distribution of carbon, oxygen, and nitrogen across the carbon matrix, confirming the successful incorporation and uniform dispersion of these elements after activation. A detectable potassium signal, originating from residual K species remaining after KOH activation. These heteroatoms and trace alkali residues contribute to the surface's basic character, which may enhance its affinity for CO_2_. Furthermore, the semi-quantitative EDX analysis shows that Af-K1 is primarily composed of carbon (90.81 ± 1.43 wt%), with moderate amounts of oxygen (7.72 ± 1.43 wt%) and nitrogen (1.39 ± 0.27 wt%), and only trace levels of potassium (0.05 ± 0.01 wt%). While STEM-EDX confirms the presence and spatial distribution of these elements, the identification of oxygen- and nitrogen-containing surface functionalities is supported by the FTIR and XPS analyses. The homogeneous elemental distribution demonstrates the chemical uniformity and activation effectiveness, consistent with STEM-EDX results showing uniform phase coverage across the carbon surface.

### 3.2. CO_2_ uptake in activated Af-biochar

**Fig. 8:**
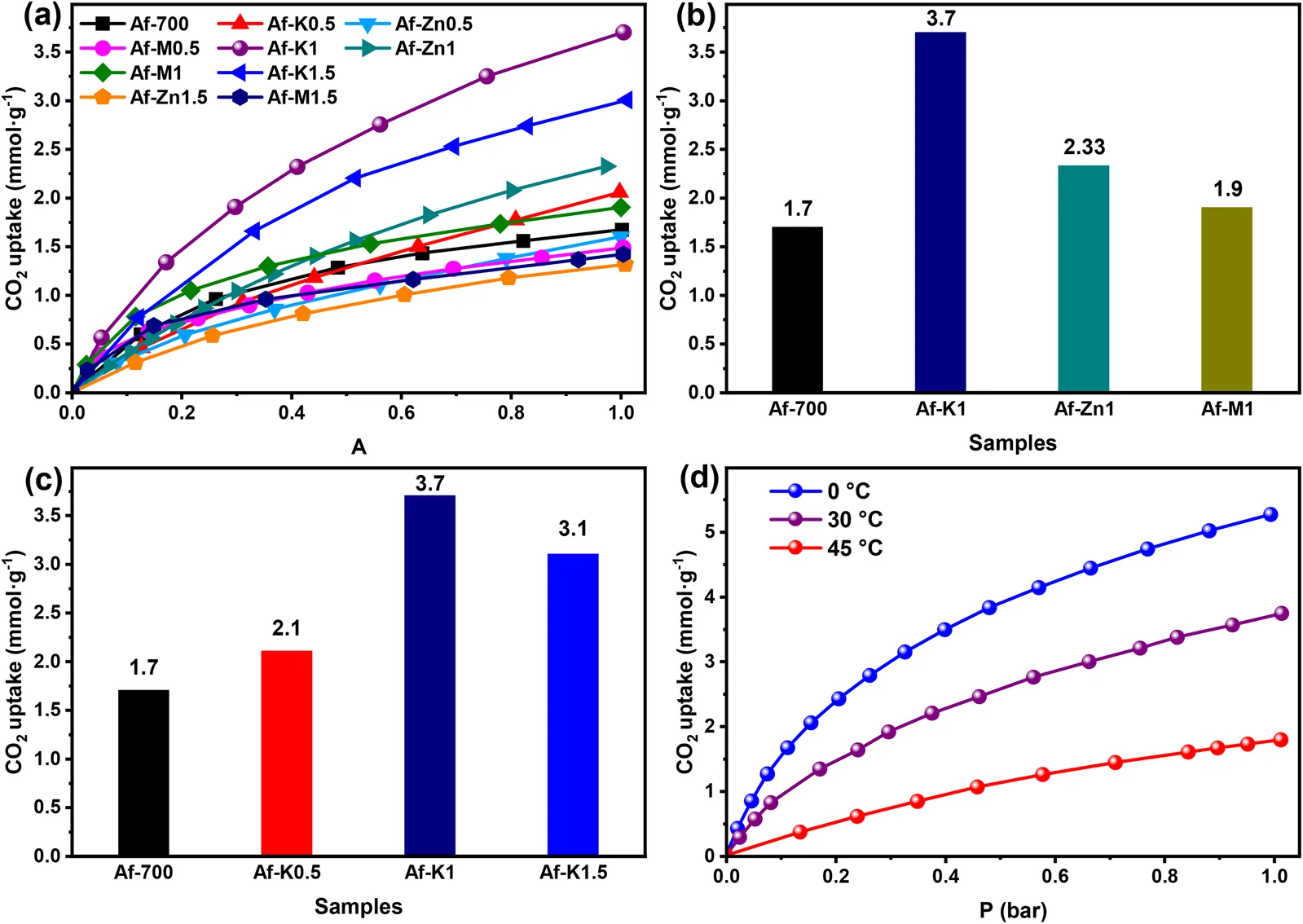
(a) CO_2_ adsorption isotherms of the Af-derived activated biochar at 30 °C, (b) different activation agents with a ratio of 1, (c) different KOH ratios, and (d) different temperatures of Af-K1.

The CO_2_ adsorption capacity of Af-derived activated biochar samples prepared in one step was evaluated within a pressure range of 0–1 bar at 30 °C. CO_2_ adsorption on porous carbons follows the Langmuir model at low pressures, where monolayer coverage dominates, and physical adsorption mechanisms prevail due to van der Waals forces and electrostatic interactions.^64^ Additionally, the effects of the active agent type, their ratio, and Af-K1 were tested at various temperatures (0, 30, and 45 °C). The results are summarized in Fig. 8, which provides a comprehensive overview of the findings: Fig. 8a displays the results of the sample evaluations, highlighting the influence of active agents and ratios on CO_2_ uptake. Af-700, the reference sample obtained from pyrolysis at 700 °C without any activating agent, exhibited a CO_2_ adsorption capacity of 1.67 mmol g^−1^, representing the intrinsic adsorption capacity of thermally treated Alfa fibers in the absence of chemical activation. Despite its very low surface area, this appreciable uptake is attributed to the abundant oxygen-containing functional groups present in the non-activated biochar, which provide polar and basic sites that interact favorably with CO_2_ molecules. This indicates that, for the non-activated sample, CO_2_ adsorption is governed primarily by surface chemistry rather than by pore structure.^65^ When biochar is modified with KOH, melamine, and ZnCl_2_ at different ratios, the adsorption capacity increases, with the maximum observed at a ratio of 1. Fig. 8b illustrates the effect of active agent type (ratio of 1) on CO_2_ capture. The CO_2_ uptake capacities of the samples are as follows: Af-700 (1.67 mmol g^−1^), Af-K1 (3.70 mmol g^−1^), Af-Zn1 (2.33 mmol g^−1^), and Af-M1 (1.90 mmol g^−1^). The superior performance of KOH-activated samples is due to their high micropore volume and narrow pore-size distribution, which maximize the overlap of adsorption potentials from opposing pore walls, creating enhanced adsorption sites for CO_2_ molecules.^66^ The enhanced CO_2_ uptake of Af-K1 compared to Af-700 can be attributed to the activation of Af with KOH, which introduces a high density of micropores and surface functional groups, thereby providing abundant sites for CO2 adsorption.^39^ The modifications with ZnCl_2_ (Af-Zn1) also increase CO_2_ uptake, as ZnCl_2_ promotes the development of porous structures, as shown in the SEM image (Fig. 5) and confirmed by BET results (Fig. 4). Melamine activation (Af-M1) introduces nitrogen functionality into the carbon structure, thereby improving CO_2_ uptake by creating strong adsorption sites through nitrogen–carbon interactions.^67^ Nitrogen-containing functional groups have been shown to enhance CO_2_ uptake due to increased polarity and interactions with CO_2_ molecules.^68^ Melamine provides pyridinic and pyrrolic nitrogen functionalities that act as Lewis base sites due to localized or partially delocalized electron-donating pairs, which interact with the electrophilic carbon center of CO_2_, a Lewis acid, through electron donation, thereby enhancing physisorption *via* acid–base mechanisms.^69^Fig. 8c presents the results of Af activated with KOH (Af-K0.5, Af-K1, and Af-K1.5), meaning the effect of the ratio on CO_2_ uptake. CO_2_ uptake showed significant improvement compared to Af-700, with capacities of 2.06, 3.70, and 3.01 mmol g^−1^, respectively. The optimal CO_2_ adsorption at intermediate KOH ratios reflects the trade-off between micropore development and pore widening. Excessive activation can convert micropores into mesopores, reducing CO_2_ adsorption efficiency because CO_2_ molecules (kinetic diameter 3.3 Å) are preferentially adsorbed in narrow micropores.^70^ KOH activation creates a well-developed microporous and mesoporous structure, which enhances CO_2_ adsorption. Increasing the KOH ratio from 0.5 to 1.5 further improves CO_2_ uptake, with the highest value (3.70 mmol g^−1^) achieved at a ratio of 1. The additional KOH promotes pore development, resulting in a balanced structure of micro- and mesopores, ideal for CO_2_ capture. Fig. 8d demonstrates the effect of temperature on the CO_2_ uptake of KOH-activated Af at a 1 : 1 ratio. The results show that CO_2_ adsorption increases as temperature decreases, with the highest CO_2_ uptake observed at 0 °C, followed by 30 °C and 45 °C, at values of 5.26, 3.70, and 1.78 mmol g^−1^, respectively. The sharp decrease in CO_2_ uptake with increasing temperature reflects the exothermic nature of physical adsorption, in which higher thermal energy disrupts the weak intermolecular forces between CO_2_ and the carbon surface.^69^ This behavior aligns with findings in the literature, such as those by Zhao *et al.*^71^ and Nandi *et al.*,^61^ which reports that CO_2_ adsorption is more efficient at lower temperatures due to stronger interactions between CO_2_ and the adsorbent; however, temperature plays a crucial role, as expected.

**Fig. 9:**
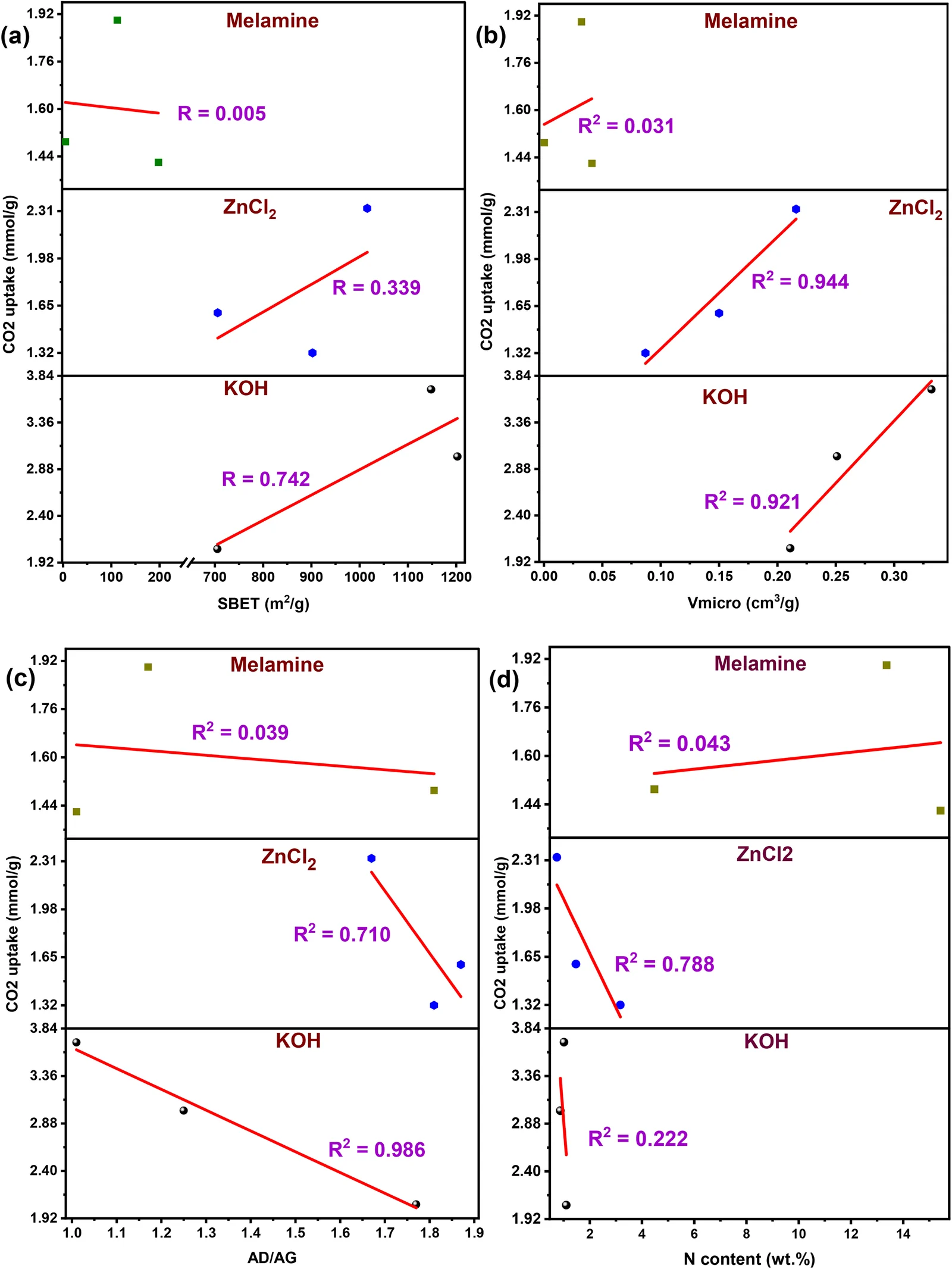
Correlation analysis between the CO_2_ adsorption capacity measured at 30 °C and 1 bar for the Af-derived activated biochars and (a) BET surface area, (b) micropore volume, (c) Raman *A*_D_/*A*_G_ ratio, and (d) nitrogen content determined by CHNS elemental analysis.

Fig. 9 presents a quantitative correlation analysis between the CO_2_ adsorption capacity and the key textural and structural properties of the prepared biochars. As shown in Fig. 9a, the CO_2_ adsorption capacity generally increases with increasing BET surface area, although the strength of the correlation depends on the activating agent. In contrast, a much stronger relationship is observed in Fig. 9b, where CO_2_ uptake correlates closely with the micropore volume, particularly for the KOH- and ZnCl_2_-activated samples (*R*^2^ = 0.921 and 0.944, respectively). These results confirm that the development of microporosity is the primary factor governing CO_2_ adsorption under near-ambient conditions. Fig. 9c shows the relationship between the Raman *A*_D_/*A*_G_ ratio and CO_2_ uptake. For the KOH-activated samples, the high correlation (*R*^2^ = 0.986) suggests that an appropriate degree of structural disorder favors the formation of adsorption sites and enhances CO_2_ uptake, whereas weaker correlations were observed for the melamine- and ZnCl_2_-activated samples. The relationship between CO_2_ adsorption capacity and nitrogen content determined by CHNS elemental analysis is presented in Fig. 9d. Compared with the textural parameters, nitrogen content exhibits relatively weak or moderate correlations with CO_2_ uptake, indicating that although nitrogen functionalities may contribute to CO_2_ adsorption, their influence is secondary to that of micropore development. Additional correlation analyses involving the micropore surface area, total pore volume, and average pore size are provided in Fig. S4. Among these parameters, the micropore surface area exhibits a very strong positive correlation with CO_2_ uptake for both the KOH- and ZnCl_2_-activated samples (*R*^2^ = 0.997 and 0.999, respectively), further confirming the dominant role of microporosity in controlling CO_2_ adsorption. In contrast, total pore volume and average pore size show weaker or less consistent correlations, indicating that the overall pore volume and larger pores contribute less significantly to CO_2_ adsorption than well-developed microporous structures. Overall, these quantitative analyses demonstrate that micropore volume is the dominant parameter governing the CO_2_ adsorption performance of the prepared biochars, while structural disorder and nitrogen content make additional contributions, depending on the activation strategy.

Furthermore, three equilibrium isotherm models; Langmuir, Freundlich, and Redlich–Peterson were applied to describe adsorption kinetics for captured CO_2_ on biochar samples (Fig. S5), with fitting parameters summarized in Table 3. The Redlich–Peterson model, which integrates features of both Langmuir and Freundlich models through the *β*_RP_ parameter, provided well-accepted fits for all samples (*R*^2^ = 0.99), confirming its suitability for heterogeneous surfaces. Referenced sample, Af-700 exhibited the lowest *q*_max_ (2.23 mmol g^−1^), consistent with its limited porosity; the Redlich–Peterson fit (*β*_RP_ ≈ 0.90) suggests predominantly Langmuir-type adsorption with moderate heterogeneity. KOH-activated samples Af-K1 and Af-K1.5 showed the highest CO_2_ capacities (3.7 and 3.01 mmol g^−1^, respectively). Both samples showed excellent agreement with the Langmuir model (*R*^2^ = 0.99), while the Redlich–Peterson model yielded *β*_RP_ values of 0.70 and 1.00, respectively. These results indicate predominantly monolayer adsorption on relatively homogeneous adsorption sites, contributing to the high CO_2_ adsorption performance of Af-K1. ZnCl_2_-activated Af-Zn1 (*q*_max_ = 3.41 mmol g^−1^) also showed a good Redlich–Peterson fit (*β*_RP_ = 1), revealing a hybrid adsorption behavior consistent with its narrow microporosity from template-driven activation. However, the melamine-modified sample (Af-M1) (*q*_max_ = 2.30 mmol g^−1^) displayed *β*_RP_ ≈ 0.74, indicating a shift toward Freundlich-type non-ideal adsorption due to nitrogen functionalities creating a wider distribution of adsorption energies. In all cases, the Freundlich model confirms that CO_2_ adsorption is favored (*n*_F_ > 1) *via* physical interactions. The consistently high *R*^2^ (≈0.99) across all models highlights the complexity of CO_2_ adsorption, with no single model emerging as the dominant mechanism. Overall, the Redlich–Peterson model demonstrates that KOH activation generates the most energetically diverse and efficient adsorption sites, thereby explaining the superior performance of Af-K1, whereas ZnCl_2_ and melamine treatments create more uniform or chemically selective surfaces, which affect CO_2_ uptake.

**Table 3:** Isotherm fitting parameters for CO_2_ adsorption on Af-derived activated biochar samples

Samples	*q*_exp_ (mmol g^−1^)	Langmuir			Freundlich			Redlich–Peterson			
		*q*_max_ (mmol g^−1^)	*k*_L_ (bar^−1^)	*R* ^2^	*k*_F_ (mmol g^−1^ bar^−1/*n*^)	*n*_F_	*R* ^2^	*k*_RP_ (mmol g^−1^ bar^−1^)	*A*_RP_ (bar^−*β*_RP_^)	*β*_RP_	*R* ^2^
Af-700	1.67	2.23	2.88	0.99	1.71	2.21	0.98	7.38	3.43	0.9	0.99
Af-M0.5	1.49	1.86	3.2	0.98	1.49	2.25	0.99	191.22	127.7	0.56	0.99
Af-Zn0.5	1.6	2.92	1.15	0.99	1.95	1.56	0.99	11.51	6.22	0.44	0.99
Af-K0.5	2.06	4.51	0.82	0.99	2.06	1.44	0.99	6.69	2.26	0.5	0.99
Af-M1	1.9	2.3	3.96	0.99	1.95	2.31	0.98	16.82	7.89	0.76	0.99
Af-Zn1	2.33	5.16	0.84	0.99	2.45	0.73	0.98	4.05	0.73	1	0.99
Af-K1	3.7	5.9	1.62	0.99	3.78	1.73	0.99	14.11	2.81	0.7	0.99
Af-M1.5	1.42	1.7	4.12	0.99	1.43	2.35	0.99	14.46	9.32	0.74	0.99
Af-Zn1.5	1.32	2.32	1.28	0.99	1.34	1.62	0.99	3.35	1.55	0.85	0.99
Af-K1.5	3.01	4.91	1.54	0.99	3.06	1.73	0.98	7.56	1.54	1	0.99

The values in Table 4 are plotted in Fig. S5 for Af-K1 at 0, 30, and 45 °C to demonstrate the effect of temperature on the adsorption mechanism. Isotherm models display good agreement with the experimental data (*R*^2^ = 0.99), confirming that Af-K1 contains both uniform and heterogeneous adsorption sites. At 0 °C, Af-K1 exhibits its highest adsorption capacity (*q*_max_ = 7.26 mmol g^−1^) and strongest affinity (*k*_L_ = 2.42 bar^−1^), indicating highly favorable CO_2_ uptake at low temperature. The corresponding Freundlich parameters (*k*_F_ = 5.46, *n*_F_ = 1.87) and the Redlich–Peterson exponent (*β*_RP_ = 0.76) further support a mixed adsorption mechanism dominated by strong interactions on high-energy sites. At an increased temperature of 30 °C, *q*_max_ decreases to 5.86 mmol g^−1^ along with reductions in *k*_L_ and *k*_F_, reflecting the expected decline in adsorption affinity for physisorption systems; *β*_RP_ (0.66) suggests slightly increased surface heterogeneity, and at 45 °C, adsorption becomes less favorable (*q*_max_ = 4.38 mmol g^−1^), with lower *k*_L_ and *k*_F_ values and an *n*_F_ approaching unity (1.36), indicating weaker interactions and reduced site energy. The increase in *β*_RP_ to 1 indicates a deviation from an ideal monolayer or purely heterogeneous adsorption, likely due to reduced interactions in micropores at elevated temperatures. The temperature-dependent declines in *q*_max_ and affinity constants indicate that CO_2_ adsorption on Af-K1 is exothermic and governed by physisorption, while combined model fits reveal coexisting uniform microporous regions.

**Table 4:** Model fitting of isothermal adsorption for Af-K1 at different temperatures

*T* (°C)	Langmuir			Freundlich			Redlich–Peterson			
	*q*_max_ (mmol g^−1^)	*k*_L_ (bar^−1^)	*R* ^2^	*k*_F_ (mmol g^−1^ bar^−1/*n*^)	*n*_F_	*R* ^2^	*k*_RP_ (mmol g^−1^ bar^−1^)	*A*_RP_ (bar^−*β*_RP_^)	*β*_RP_	*R* ^2^
0	7.26	2.42	0.99	5.46	1.87	0.99	25.17	3.77	0.76	0.99
30	5.86	1.63	0.99	3.78	1.69	0.99	15.29	3.13	0.66	0.99
45	4.38	1.01	0.99	1.8	1.36	0.99	2.97	0.67	1	0.99

### 3.3. Af-K1 profiling

**Fig. 10:**
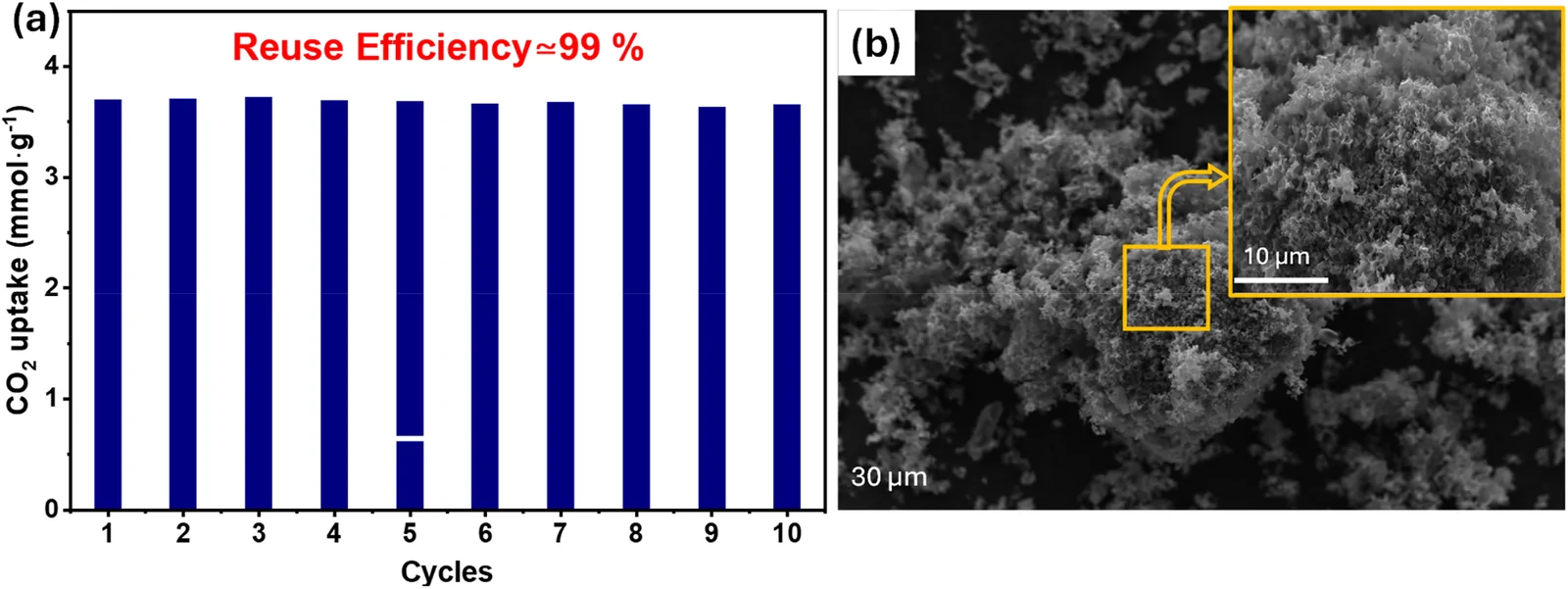
(a) CO_2_ adsorption capacity of Af-K1 over ten adsorption–desorption cycles at 30 °C and 1 bar; and (b) SEM images of Af-K1 after the tenth cycle.

Ten consecutive CO_2_ adsorption–desorption cycles were performed to evaluate the cyclic stability and reusability of Af-K1 under the laboratory conditions investigated. As shown in Fig. 10a, the CO_2_ uptake decreased only slightly from 3.70 mmol g^−1^ in the first cycle to 3.66 mmol g^−1^ in the tenth cycle, corresponding to a capacity loss of approximately 1% and a retention of ∼99% of the initial adsorption capacity. These results demonstrate the high cyclic stability of Af-K1 and are consistent with the performance of KOH-activated biochars reported in the literature.^72^ Furthermore, SEM images of Af-K1 before adsorption (Fig. 5) and after ten adsorption–desorption cycles (Fig. 10b) revealed no noticeable surface degradation or structural collapse. The irregular surface roughness and well-preserved porous morphology, consistent with the observations of Monteagudo *et al.*,^45^ indicate that the pore structure remained stable throughout the cycling test. Although these results confirm excellent short-term stability under dry, pure CO_2_ adsorption and vacuum regeneration conditions, further long-term evaluation under humid and CO_2_/N_2_ mixed-gas conditions is required to assess the adsorbent's practical stability.

To assess the CO_2_ adsorption performance of Af-K1, its adsorption capacity was compared with that of one-step activated biomass-derived carbons from the literature, as summarized in Table S2. Af-K1 was tested at a higher temperature (30 °C) and 1 bar. Yet, it still achieved a CO_2_ uptake of 3.70 mmol g^−1^, outperforming BBC-KOH-1 : 1 (biochar from bamboo charcoal activated by KOH (1 : 1) at 700 °C, 1 h) (3.49 mmol g^−1^) reported by Zhang *et al.*,^11^ KLB2 (*Arundo donax* activated by KOH (1 : 2) at 600 °C, 2 h in one step.) (3.6 mmol g^−1^) prepared by Singh *et al.*,^39^ and AC-700 (fern leaves activated by KOH (1 : 1) under flow of CO_2_ at 700 °C, 1 h) (3.58 mmol g^−1^) developed by Serafin *et al.*,^73^ seaweed-derived biochar prepared by Ding *et al.*^17^ (KOH (1 : 1), 800 °C, 2 h), which exhibited a CO_2_ uptake of 1.05 mmol g^−1^ at 25 °C under 12% CO_2_, and peanut shell-derived porous carbon reported by Wang *et al.*,^18^ which achieved a CO_2_ uptake of 2.41 mmol g^−1^ at 20 °C under 15% CO_2_ using a one-step K_2_CO_3_ activation strategy. This result indicates that Af-K1 retains a high CO_2_ adsorption capacity at elevated temperatures, despite the general decrease in physisorption capacity with increasing temperature. Among the materials compared, SD2700 (hydrochar from sawdust activated with KOH (1 : 2) and carbonized at 700 °C for 1 h), reported by Balahmar *et al.*,^21^ exhibited the highest uptake of 4.6 mmol g^−1^. Which, however, was prepared through a more energy-intensive and complex two-step activation process, involving initial carbonization followed by chemical activation with KOH at a high ratio (2 : 1), and subsequent heat treatment at 700 °C. In our instance, Af-K1 was created using a one-step activation method, which made it easier to scale and more energy-efficient. Af-K1 is thus a very promising candidate for sustainable and economical CO_2_ capture, especially in near-ambient conditions, due to its high performance, low energy consumption, and simple preparation.

Table 5 summarizes the thermodynamic parameters (Δ*H*°, Δ*G*°, and Δ*S*°) of the Af-K1 sample at different temperatures by using van't Hoff's eqn (5)–(7) to reveal important insights into the CO_2_ adsorption process. The negative Δ*G*° values at all temperatures (0, 30, and 45 °C) indicate that the CO_2_ adsorption process is spontaneous, with Δ*G* ° becoming less negative as the temperature increases. This suggests that while the adsorption remains thermodynamically favorable, its spontaneity decreases at higher temperatures. The negative Δ*S* ° value (−0.04 kJ mol^−1^ K^−1^) implies a decrease in entropy, indicating that the adsorption leads to a more ordered system, where the adsorbate molecules become more structured upon interaction with the adsorbent. Additionally, the negative Δ*H*° value (−13.189 kJ mol^−1^) confirms that the adsorption is exothermic, releasing energy as CO_2_ molecules are adsorbed onto the surface. This energy release occurs because the formation of intermolecular interactions between CO_2_ and the active sites on the carbon surface is energetically more favorable than when CO_2_ molecules remain in the gas phase.^38^ Overall, the process is energetically favorable, but the increase in temperature reduces its thermodynamic favorability, primarily due to the negative entropy change. This suggests that the process is more favorable at lower temperatures, with heat being released as CO_2_ molecules interact with the adsorbent. The isosteric heat of adsorption 
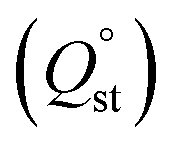
 for CO_2_ on Af-K1 was determined using the Clausius–Clapeyron equation from adsorption isotherms measured at 0, 30, and 45 °C. As summarized in Table S3, the calculated 
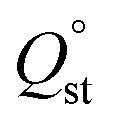
 values remained nearly constant over the investigated CO_2_ loading range, decreasing slightly from 31.9 kJ mol^−1^ at a CO_2_ loading of 0.40 mmol g^−1^ to 29.7 kJ mol^−1^ at loadings of 1.20–1.30 mmol g^−1^, before increasing marginally to 30.1 kJ mol^−1^ at 1.70 mmol g^−1^. The overall variation (∼2 kJ mol^−1^) is considerably smaller than the associated uncertainty (±12–15 kJ mol^−1^), indicating that the adsorption energetics are essentially independent of CO_2_ loading within the experimental uncertainty. This behavior suggests a relatively uniform distribution of adsorption sites in the KOH-activated biochar. Furthermore, the thermodynamic parameters indicate that CO_2_ adsorption on Af-K1 is spontaneous and exothermic, with the adsorption process being more favorable at lower temperatures.

**Table 5:** Thermodynamic parameters for CO_2_ adsorption on Af-K1 biochar at different temperatures, Gibbs free energy change (Δ*G*°), entropy change (Δ*S*°), and enthalpy change (Δ*H*°)

Temperature (°C)	Thermodynamics parameters		
	Δ*G*° (kJ mol^−1^)	Δ*S*° (kJ mol^−1^ K^−1^)	Δ*H*° (kJ mol^−1^)
0	−2.11	−0.04	−13.19
30	−0.89		
45	−0.28		

### 3.4. CO_2_/N_2_ selectivity of Af-K1

**Fig. 11:**
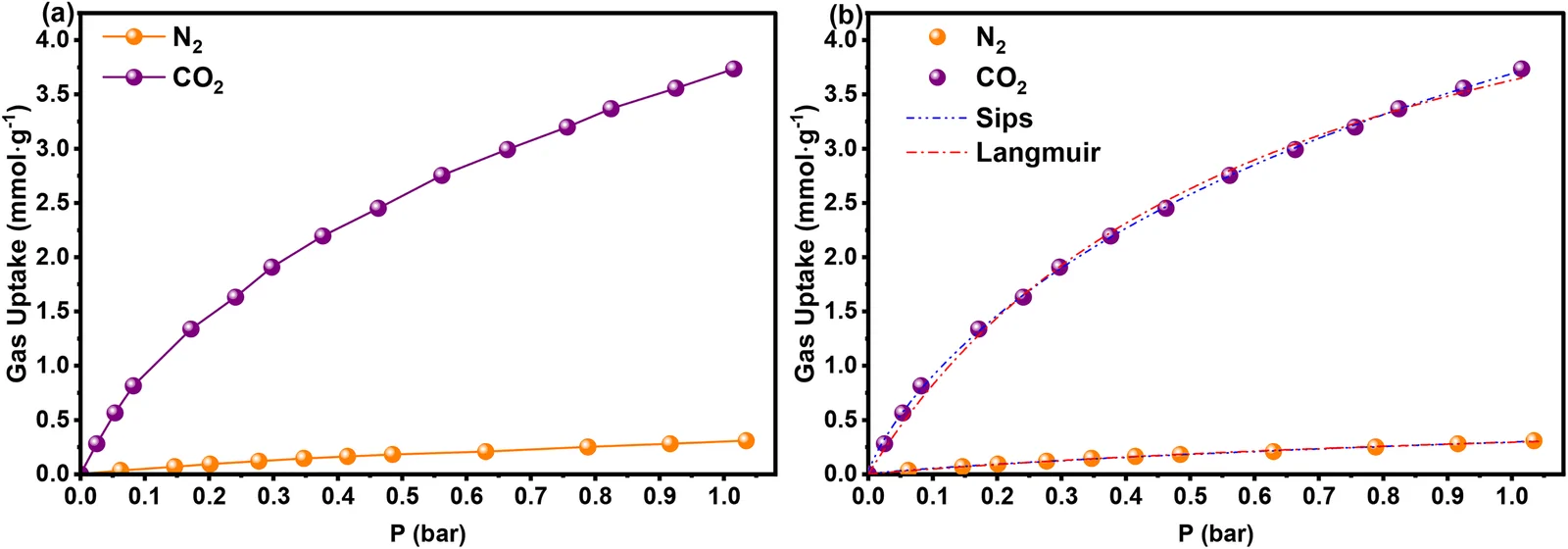
(a) Comparison of CO_2_ and N_2_ uptake at 30 °C for sample Af-K1 and (b) corresponding nonlinear Langmuir and Sips fittings applied to the experimental data.

To evaluate the adsorption equilibrium of CO_2_ and N_2_ on Af-K1, the experimental adsorption isotherms were fitted using the Langmuir and Sips models (Fig. 11 and Table 6). Both models provided an excellent fit to the experimental data, with coefficients of determination (*R*^2^) exceeding 0.997. Nevertheless, the Sips model exhibited a slightly superior fit, yielding *R*^2^ values of 0.999 for CO_2_ and 0.998 for N_2_. Furthermore, the Sips heterogeneity parameter (*n*) was lower than unity for both gases, indicating adsorption on an energetically heterogeneous surface, which is consistent with the heterogeneous pore structure and surface chemistry introduced by KOH activation. Consequently, the Sips model was selected to describe the adsorption equilibrium and subsequently employed to calculate the CO_2_/N_2_ selectivity using Ideal Adsorbed Solution Theory (IAST).

**Table 6:** Langmuir and Sips isotherm parameters obtained by fitting the experimental CO_2_ and N_2_ adsorption isotherms of Af-K1 at 30 °C

Gas	Model	*q*_m_ (mmol g^−1^)	*k*_L_ (bar^−1^) *k*_S_ (bar^−1^)	*n*	*R* ^2^
CO_2_	Langmuir	5.86	1.62	—	0.997
	Sips	8.92	0.65	0.8	0.999
N_2_	Langmuir	0.7	0.73	—	0.997
	Sips	1.48	0.19	0.83	0.998

We assessed the CO_2_/N_2_ selectivity of Af-K1 by comparing the amounts of CO_2_ and N_2_ adsorbed at 30 °C and 1 bar (Fig. 11a). At 1 bar, Af-K1 stored 3.7 mmol per g CO_2_, far exceeding the N_2_ uptake of 0.30 mmol g^−1^, corresponding to an equilibrium CO_2_/N_2_ uptake ratio of 12. We further estimated selectivity under simulated post-combustion flue-gas conditions, in which CO_2_ comprises *ca.* 15% of the stream, and the balance is mainly N_2_, by comparing CO_2_ uptake at 0.15 bar with N_2_ uptake at 0.85 bar. On this basis, using *S* = [*n*(CO_2_)·*p*(N_2_)]/[*n*(N_2_)·*p*(CO_2_)] with *n*(CO_2_) and *n*(N_2_) evaluated at 0.15 and 0.85 bar, respectively, a selectivity of 25 was obtained, comparable to values reported for carbon materials.^21^

To provide a more rigorous, thermodynamically consistent estimate that accounts for competitive co-adsorption, the CO_2_/N_2_ selectivity was also computed using Ideal Adsorbed Solution Theory (IAST), applying the Sips model to both isotherms. For a 15 : 85 CO_2_/N_2_ mixture at 1 bar and 30 °C, IAST predicted a selectivity of 58, which is higher than the simple partial pressure estimates because it accounts for the suppression of N_2_ uptake by co-adsorbed CO_2_. The selectivity increased with total pressure and with CO_2_ mole fraction (Fig. S7), and Langmuir- and Sips-based calculations agreed to within a few units, confirming that the value is not an artifact of the isotherm model.

The selectivity was further estimated under direct-air-capture conditions (400 ppm CO_2_ in N_2_, 1 bar, 30 °C). As shown in Fig. S8a, this CO_2_ partial pressure (4 × 10^−4^ bar) lies well below the experimentally measured range (shaded region), so the isotherms were extrapolated using the fitted models. Under these conditions, IAST predicted a CO_2_/N_2_ selectivity of 24–41 (Langmuir and Sips models, respectively; Fig. S8b), corresponding to a CO_2_ uptake of *ca.* 0.003–0.005 mmol g^−1^. As illustrated in Fig. S8b, the two models converge near the flue-gas composition but diverge increasingly towards lower CO_2_ fractions, reflecting the growing sensitivity of the predicted selectivity to the isotherm model at ultralow coverage. These values should therefore be regarded as model predictions rather than experimentally validated DAC performance, since low-pressure CO_2_ adsorption measurements below *ca.* 0.01 bar, ideally complemented by mixed-gas adsorption or breakthrough experiments under realistic DAC conditions, would be required to validate the predicted selectivity of Af-K1.

## 4. Conclusion

Af-derived biochar is significantly affected by one-step chemical activation agents (KOH, melamine, and ZnCl_2_) in evaluating CO_2_ uptake capacity. The most successful activation agent was KOH, which greatly increased the surface area and porosity of biochar, resulting in the highest CO_2_ adsorption capacity. In particular, 1 : 1 mass ratio optimized as Af-K1 outperformed other samples with a CO_2_ uptake of 3.70 mmol g^−1^, a surface area of 1148 m^2^ g^−1^, and a total pore volume of 0.509 cm^3^ g^−1^. KOH activation produced a well-developed microporous and mesoporous architecture, which contributed to the high CO_2_ adsorption capacity of Af-K1.

Conversely, KOH had a greater effect on CO_2_ adsorption than activation with melamine and ZnCl_2_, despite the latter two contributing to chemical changes and porosity. High CO_2_ uptake is further supported by the Redlich–Peterson model, which fits all samples better than the Langmuir and the Freundlich models and suggests the presence of uniform, slightly heterogeneous adsorption sites. Thermodynamic analysis indicated that CO_2_ adsorption onto Af-K1 is spontaneous and exothermic, with Δ*H*° = −13.189 kJ mol^−1^ and Δ*S*° = −0.041 kJ mol^−1^ K^−1^. Gibbs free energy (Δ*G*°) remained negative at all tested temperatures (−2.109, −0.892, and −0.283 kJ mol^−1^ at 0, 30, and 45 °C, respectively), confirming the process's favorability, particularly at lower temperatures. In addition to its high capacity, Af-K1 exhibited excellent cyclic stability under conditions investigated, remaining ∼99.0% effective even after 10 cycles, with only a ∼1.0% loss. SEM images acquired before and after cycling confirmed that the porous morphology remained intact, with no evidence of structural degradation or pore collapse. Further long-term evaluation under humid and mixed-gas conditions is required to confirm its practical stability. In addition to its adsorption capacity and structural stability, Af-K1 exhibited an IAST-predicted CO_2_/N_2_ selectivity of 58 under simulated post-combustion flue-gas conditions (15% CO_2_, 1 bar, 30 °C). Model extrapolation also predicted CO_2_/N_2_ selectivity in the range of 24–41 under direct air capture (DAC) conditions (400 ppm CO_2_). These predicted values suggest that Af-K1 may warrant further investigation for DAC. However, direct low-pressure CO_2_ adsorption and mixed-gas experiments under realistic, humid DAC conditions are required to verify this potential. Overall, these findings demonstrate that one-step KOH activation is an effective and sustainable strategy for producing high-performance biochar adsorbents for CO_2_ capture and separation.

## Author contributions

Hicham Akaya: writing – original draft, validation, investigation, data curation, visualization, methodology, formal analysis. Zineb Ouzrour: writing – original draft, validation, investigation, data curation, visualization, methodology, formal analysis. Badr El Aalami: writing – original draft, validation, investigation, data curation. Alexandre Babin: writing – original draft, validation, investigation, data curation. Hosni Idrissi: writing – review & editing, validation, investigation, data curation. Xiaotao Bi: writing – review & editing, supervision, validation, investigation, data curation. Tausif Altamash: conceptualization, writing – review & editing, validation, supervision, investigation, visualization, project management. Johan Jacquemin: conceptualization, validation, formal analysis, resources, writing – review & editing, visualization, supervision, project administration, funding acquisition.

## Conflicts of interest

The authors declare no known competing financial interests or personal relationships that could have influenced the work reported in this paper.

## Supplementary Material

RA-016-D6RA06467A-s001

## Data Availability

The data supporting this study are available from the corresponding author upon request. Supplementary information (SI): additional characterization and adsorption analyses of the Alfa fiber-derived biochars. Table S1 summarizes the elemental composition (C, H, N, S, and O) of the prepared biochar samples. Fig. S1 presents (a) FTIR spectra and (b) XRD patterns of the Af-derived activated biochars, offering insight into surface functional groups and structural ordering. The pore size distribution obtained from adsorption measurements is shown in Fig. S2. Fig. S3. STEM-EDX analysis of the ZnCl_2_-activated Alfa-fiber biochars Af-Zn0.5, Af-Zn1, and Af-Zn1.5. Table S2. Comparison of the physicochemical properties and CO_2_ adsorption performance of recently reported one-step activated biomass-derived porous carbons and the optimized Af-K1 sample prepared in this study. Fig. S4. Additional correlation analyses between the CO_2_ adsorption capacity measured at 30 °C and 1 bar for the Af-derived activated biochars and (a) micropore surface area, (b) total pore volume, and (c) average pore size. Fig. S5 displays the nonlinear fitting of CO_2_ adsorption isotherms using the Langmuir, Freundlich, and Redlich–Peterson models. Thermodynamic analysis of CO_2_ adsorption on Af-K1 is illustrated in Fig. S6, including van't Hoff plots used to determine Δ*H*° and Δ*S*°, as well as the calculated Δ*G*° values. The CO_2_ loadings selected for the calculation of the isosteric heat of adsorption 
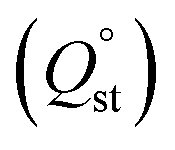
, together with the corresponding linear fitting parameters, are listed in Table S3. Finally, Fig. S7. IAST-predicted CO_2_/N_2_ selectivity using Sips/Sips and Langmuir/Langmuir adsorption models: (a) selectivity as a function of total pressure at a feed composition of 15% CO_2_/85% N_2_ and (b) selectivity as a function of CO_2_ mole fraction at 1 bar. The dashed vertical line denotes the 15% CO_2_ feed composition representative of post-combustion flue and Fig. S8. (a) CO_2_ adsorption isotherm of Af-K1 at 30 °C with Sips and Langmuir fits; the shaded region below 0.025 bar is extrapolated. (b) IAST CO_2_/N_2_ selectivity *versus* CO_2_ mole fraction at 1 bar and 30 °C, showing the flue-gas (15% CO_2_) and direct-air-capture (400 ppm CO_2_) compositions. See DOI: https://doi.org/10.1039/d6ra06467a.
