# Supplementary material for: Enhanced CO2 adsorption via one-step thermochemical activation of Alfa fiber
Source: RSC Adv. 2026 Aug 26;16(42):50576–94. doi: 10.1039/d6ra06467a (PMC13508056; doi:10.1039/d6ra06467a)
Supplement: Supplementary file 1 [file RA-016-D6RA06467A-s001.pdf]

Supplementary Information (SI) for RSC Advances.
This journal is © The Royal Society of Chemistry 2026

Electronic Supporting Information

Enhanced CO2 Adsorption via One-Step Thermochemical Activation of Alfa Fiber

Hicham Akaya[a], Zineb Ouzrour[a], Badr El Aalami[b,c], Alexandre Babin[d], Hosni Idrissi[c], Xiaotao Bi[d], Tausif Altamash[a,*], Johan Jacquemin[a,*]

[a] College of Chemical Sciences and Engineering (CCSE), Department of Materials Science, Energy and Nano-engineering (MSN), Mohammed VI Polytechnic University (UM6P), 43150 Benguerir, Morocco.

[b] High Throughput Multidisciplinary Research Laboratory (HTMR), College of Chemical Sciences and Engineering (CCSE), Mohammed VI Polytechnic University (UM6P), Lot 660, Benguerir 43150, Morocco.

[c] Institute of Mechanics, Materials and Civil Engineering, IMAP Division, UCLouvain, Place Sainte Barbe, 2, B-1348, Louvain-la-Neuve, Belgium.

[d] Chemical and Biological Engineering Department, University of British Columbia, Vancouver, British Columbia V6T 1Z3, Canada.

* ***Co-corresponding authors:***

Emails: tausif.altamash@um6p.ma; Johan.Jacquemin@um6p.ma

Table S1. Proximate and Ultimate Analysis of Alfa Fiber-Derived Biochar.

Samples	Yield (wt.%)	Proximate Analysis (wt.%)				Ultimate Analysis (wt.%)			
		Moisture	VM	FC	Ash	C	H	N	O*
Af-700	24.2	4.34	10.48	78.52	6.66	48.68	1.81	0.8	42.05
Af-M0.5	25.9	7.55	9.38	75.66	7.41	69.71	1.55	4.47	16.86
Af-Zn0.5	22.2	19.96	15.61	60.94	3.49	61.18	1.89	1.47	31.97
Af-K0.5	20.5	19.33	15.16	62.57	2.94	63.11	2.07	1.1	30.78
Af-M1	27.4	11.19	13.54	70.99	4.28	62.03	1.68	13.86	18.15
Af-Zn1	21.5	13.2	18.01	62.93	5.86	60.91	1.84	0.74	30.65
Af-K1	20.1	11.03	14.42	70.57	3.98	57.24	2.12	1.01	35.65
Af-M1.5	29.3	9.71	16.13	63.19	10.97	48.21	1.58	15.43	23.81
Af-Zn1.5	20.4	13.2	16.11	65.58	5.11	52.48	1.88	3.17	37.36
Af-K1.5	19.71	18.14	18.82	60.77	2.27	56.08	2.22	0.87	38.56

* Calculated by difference on an ash-inclusive basis with S = 0 wt.%.

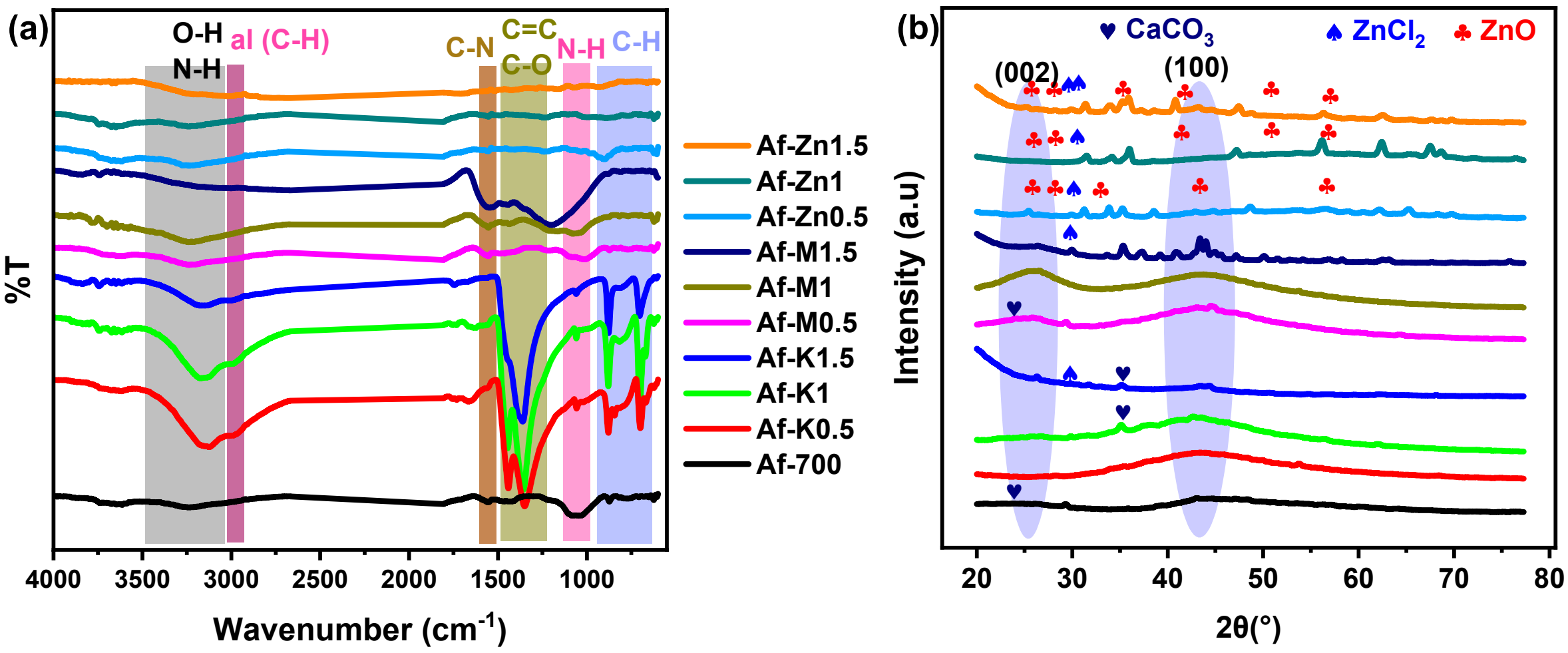

Figure S1. (a) FTIR spectra and (b) XRD pattern of Af-derived activated biochar.

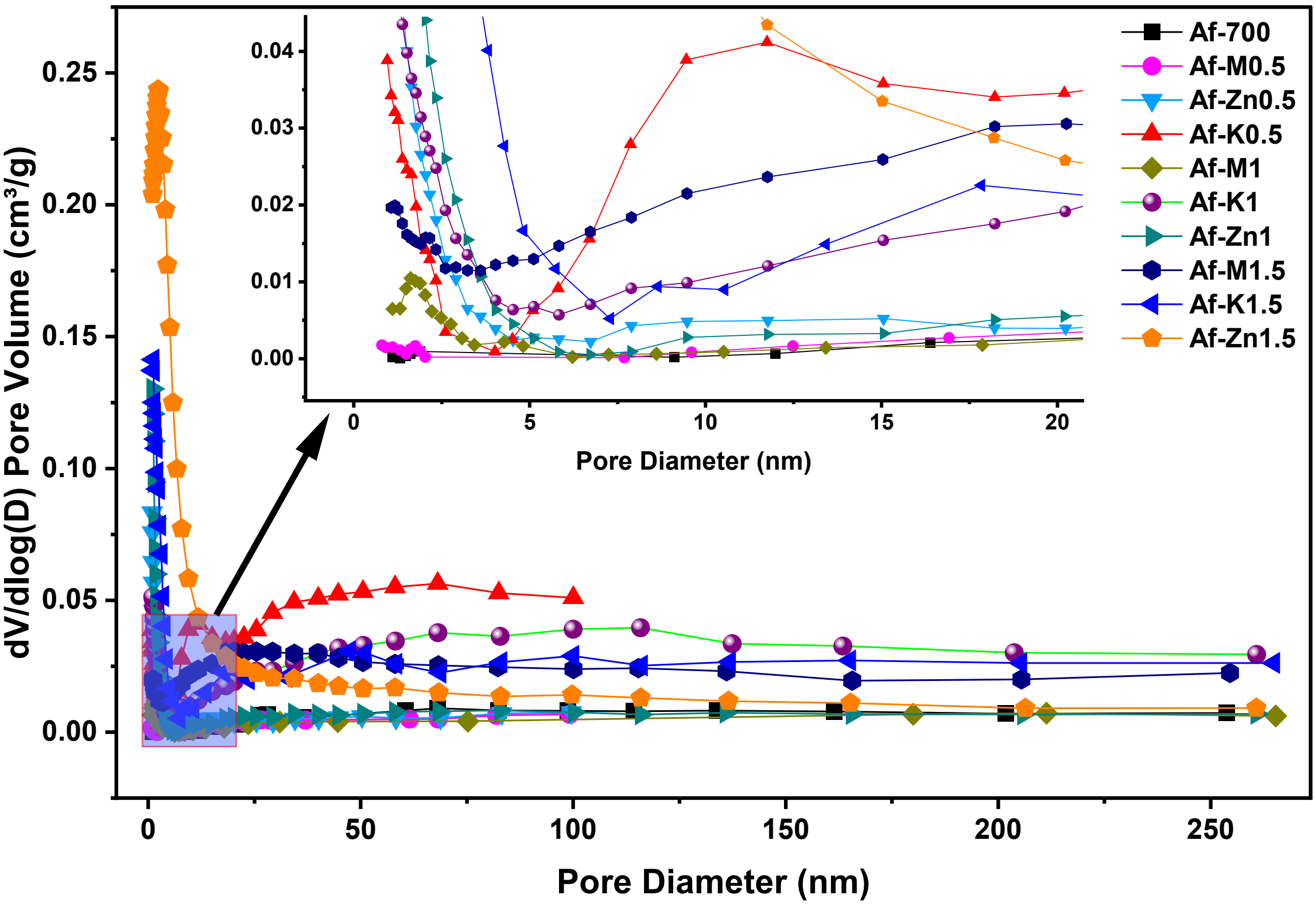

Figure S2. pore size distribution of Af-derived activated biochar.

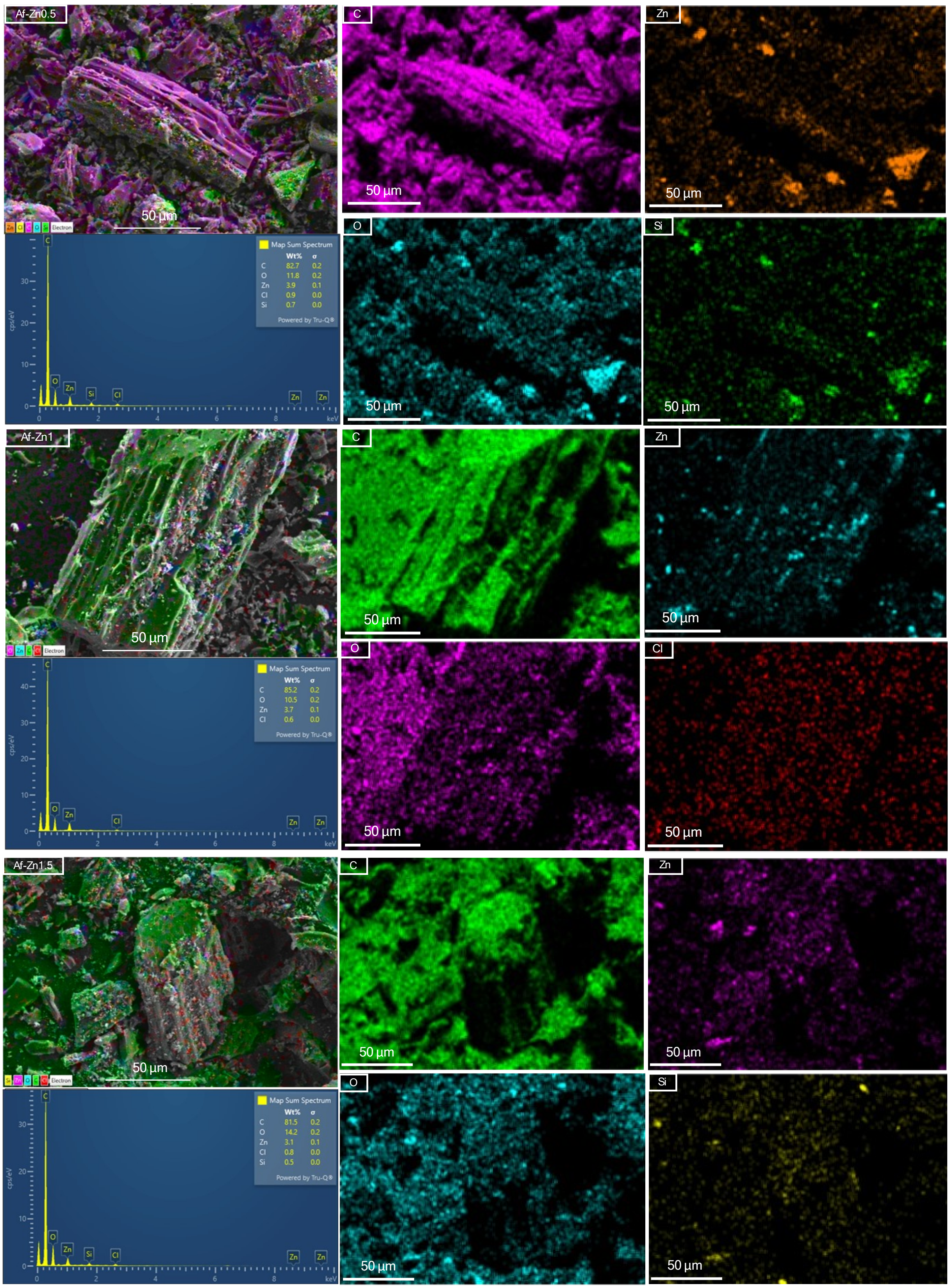

Figure S3. STEM-EDX analysis of the $ZnCl_2$-activated Alfa-fiber biochars Af-Zn0.5, Af-Zn1, and Af-Zn1.5

Table S2. Comparison of the physicochemical properties and CO_2 adsorption performance of recently reported one-step activated biomass-derived porous carbons and the optimized Af-K1 sample prepared in this study.

Biomass precursor	Activation strategy	Activation Temperature	Activator /Biomass ratio	BET (m^2/g)	Micropore volume (cm^3/g)	CO_2 uptake (mmol g^{-1})	Adsorption conditions	Ref
Arundo donax	One-step KOH	600 °C, 2 h	2:1	1122	0.50	6.30	0 °C, 1 bar	1
						3.6	25 °C, 1 abr	
Sawdust	Direct one-step KOH	800 °C, 1h	2:1	2274	0.90	4.60	25 °C, 1 bar	2
						1.3	25 °C, 0.15 bar	
Seaweed (Sargassum)	One-step KOH	800 °C, 2h	1:1	291.8	-	1.05	25 °C, 12% CO_2	3
Peanut shell	One-step KOH/K_2CO_3	750 °C, 0.5h	0.5:1	626 / 669	0.30 / 0.35	2.41	20 °C, 15% CO_2	4
Fern leaves	Simultaneous carbonization + activation (KOH + CO_2)	700 °C, 1h	1:1	1016	0.539	6.77	0 °C, 1 bar	5
						3.58	25 °C, 1 bar	
Bamboo	One-step KOH	700 °C, 1h	1:1	728	0.25	3.49	25 °C, 1 bar	6
Moroccan Alfa fiber	One-step activation (KOH, $ZnCl_2$, melamine)	700 °C, 1h	1:1	1148	0.332	5.26	0 °C, 1 bar	This work
						3.70	30 °C, 1 bar	
						1.2	30 °C, 0.15 bar	

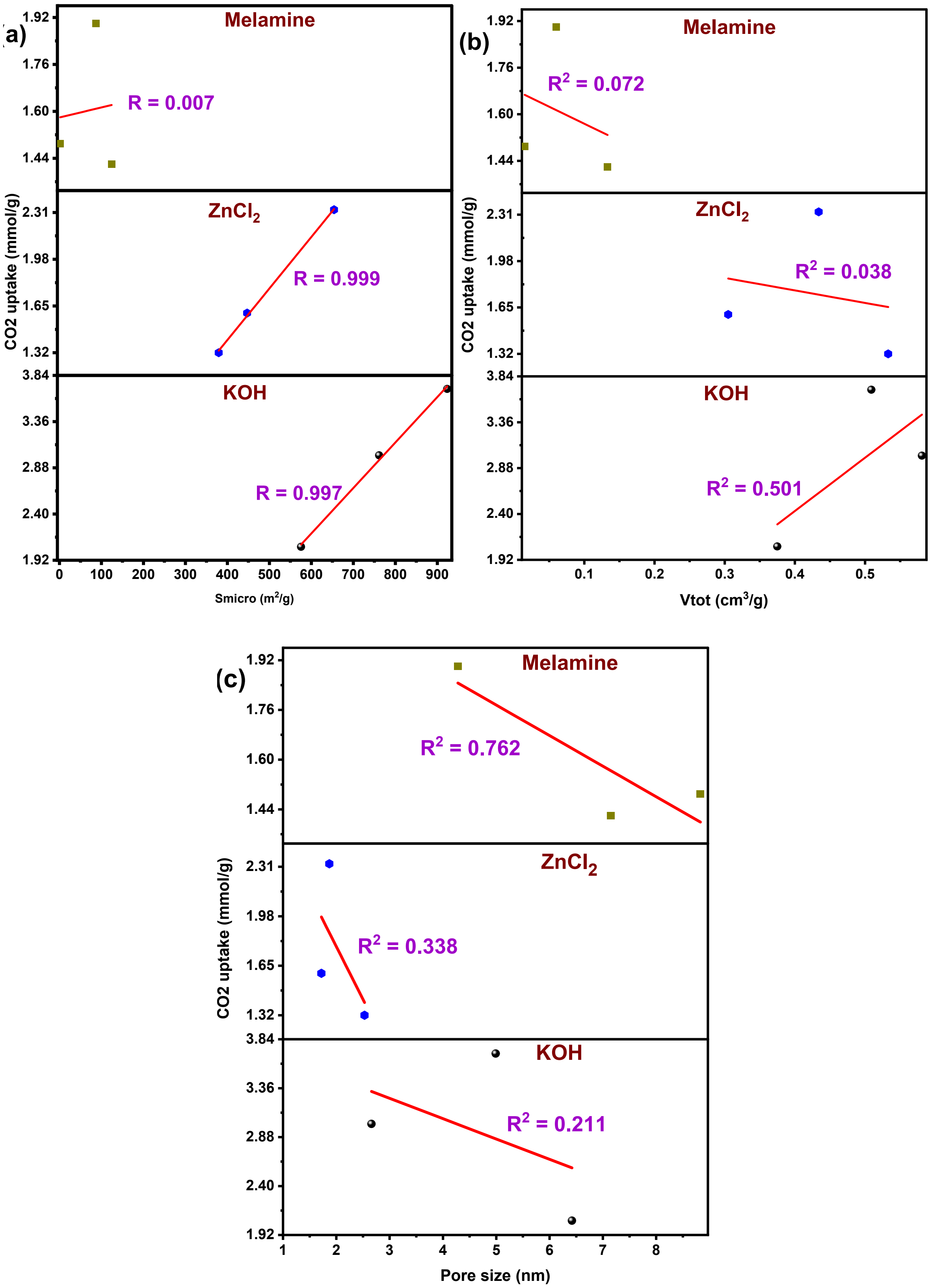

Figure S4. Additional correlation analyses between the CO_2 adsorption capacity measured at 30 °C and 1 bar for the Af-derived activated biochars and (a) micropore surface area, (b) total pore volume, and (c) average pore size.

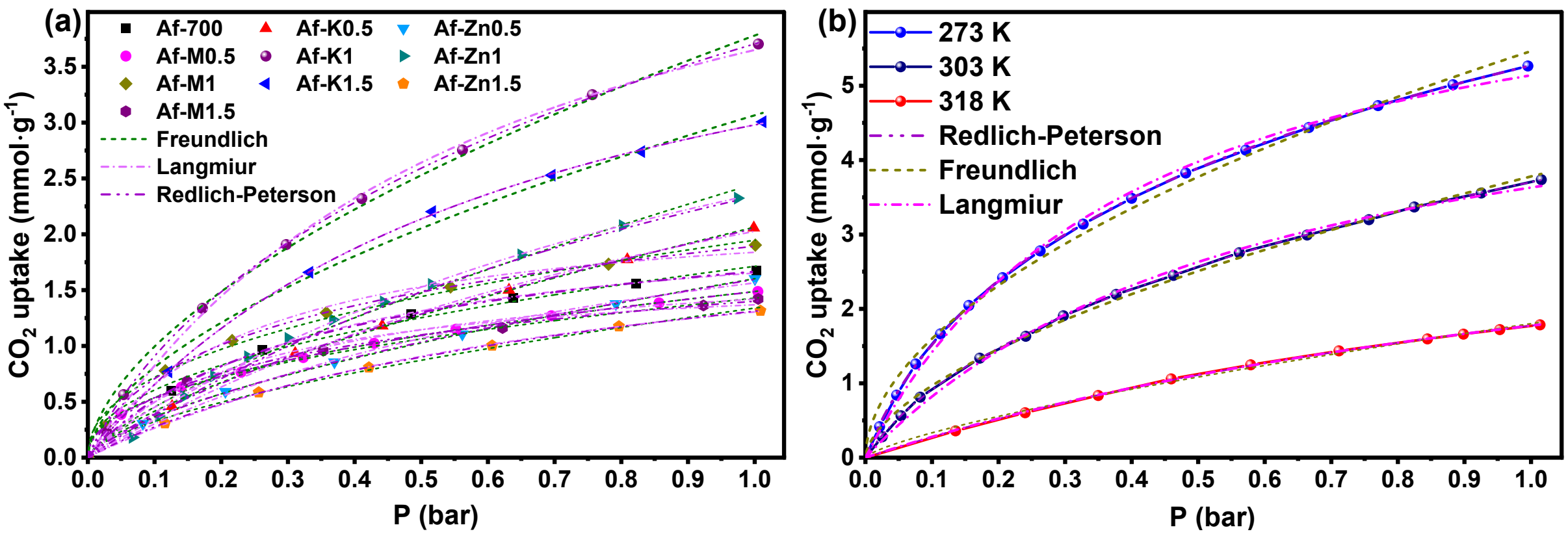

Figure S5. Nonlinear Langmuir, Freundlich, and Redlich-Peterson fittings applied to the CO_2 adsorption isotherms of the Af-derived activated biochar.

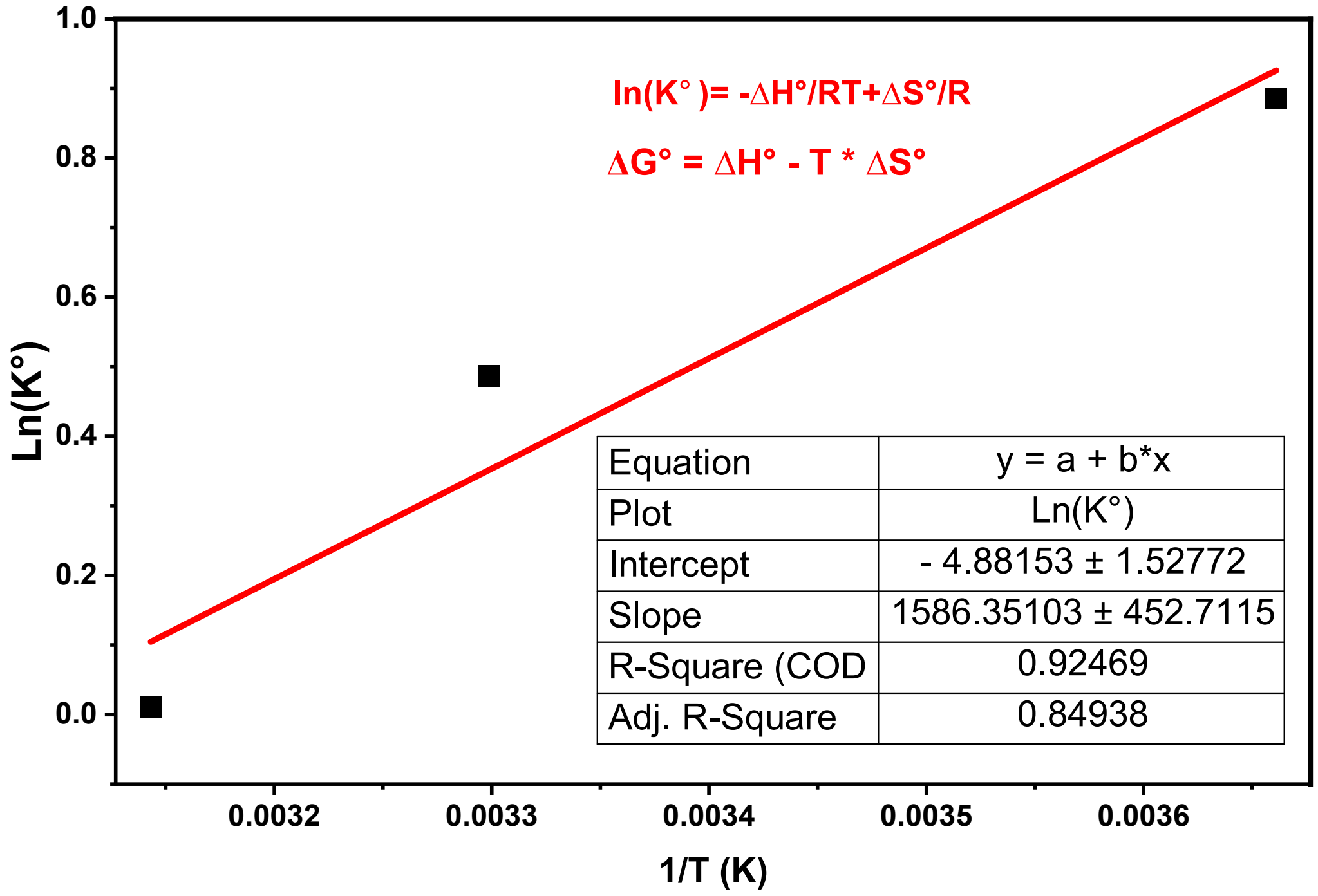

Figure S6. van't Hoff linear fitting was used to calculate the thermodynamic parameters ΔH° and ΔS°, and the ΔG° values for CO_2 adsorption on Af-K1 were also calculated.

Table S3. Isosteric heat of adsorption (Q°st) of CO_2 on the Af-K1 sample as a function of CO_2 loading, calculated using the Clausius-Clapeyron equation. Uncertainties correspond to the standard error of the Van't Hoff linear regression.

Loading ($mmol{\cdot}g^{-1}$)	Q°st ($kJ{\cdot}mol^{-1}$)	Uncertainty (± $kJ{\cdot}mol^{-1}$)
0.40	31.9	±15

0.50	31.3	±14
0.60	30.8	±14
0.70	30.5	±13
0.80	30.2	±13
0.90	30.0	±13
1.00	29.9	±12
1.10	29.8	±12
1.20	29.7	±12
1.30	29.7	±12
1.40	29.8	±12
1.50	29.9	±12
1.60	30.0	±12
1.70	30.1	±12

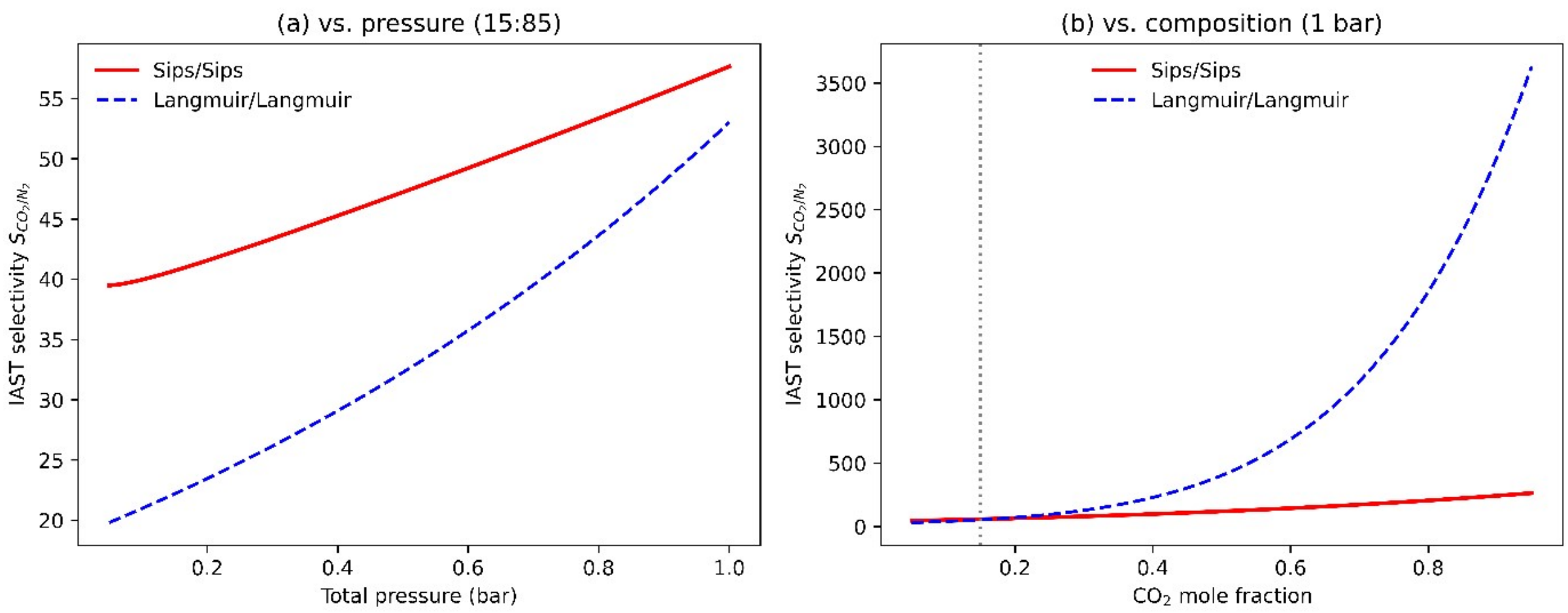

Figure S7. IAST-predicted CO_2/N_2 selectivity using Sips/Sips and Langmuir/Langmuir adsorption models: (a) selectivity as a function of total pressure at a feed composition of 15% CO_2/85% N_2 and (b) selectivity as a function of CO_2 mole fraction at 1 bar. The dashed vertical line denotes the 15% CO_2 feed composition representative of post-combustion flue gas.

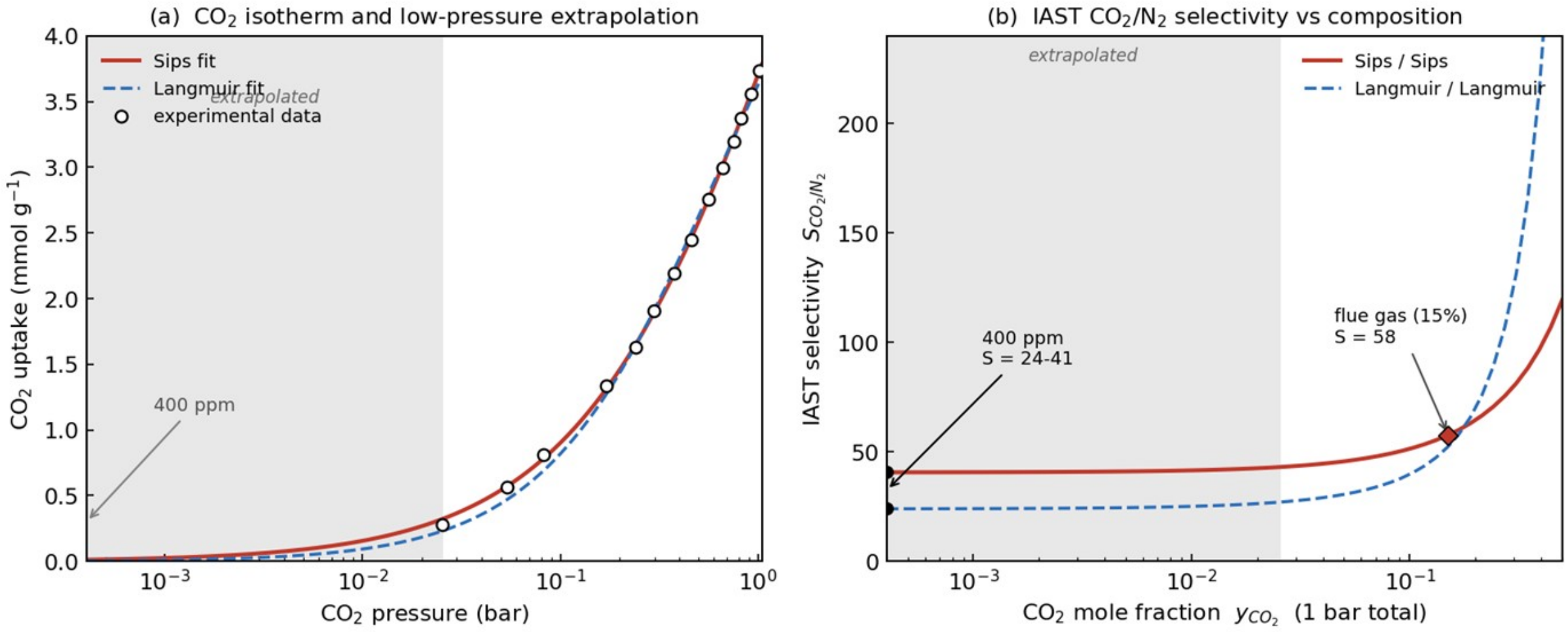

Figure S8. (a) CO_2 adsorption isotherm of Af-K1 at 30 °C with Sips and Langmuir fits; the shaded region below 0.025 bar is extrapolated. (b) IAST CO_2/N_2 selectivity versus CO_2 mole fraction at 1 bar and 30 °C, showing the flue-gas (15% CO_2) and direct-air-capture (400 ppm CO_2) compositions.
